# Synthesis and reactions of di(thiophen-2-yl)alkane diones: Cyclocondensation

**DOI:** 10.55730/1300-0527.3446

**Published:** 2022-04-28

**Authors:** Tekin ARTUNÇ, Abdullah MENZEK

**Affiliations:** Department of Chemistry, Faculty of Science, Atatürk University, Erzurum, Turkey

**Keywords:** Acylation, bromination, cyclocondensation, diketone, thiophene

## Abstract

Known 1,6-di(thiophen-2-yl)hexane-1,6-dione (**2**) and novel 1,7-di(thiophen-2-yl)heptane-1,7-dione (**4**) were obtained from the reactions of thiophene with the corresponding diacyl chlorides. Furthermore, compounds with furan and pyrrole units in place of thiophene units in compound **2** were obtained in the same way. Bromination of **2** and **4** gave bromides regioselectively. The reaction of each of the compounds **2** and **4** in HOAc medium yielded cyclocondensation products. In total, four known and eleven novel compounds were synthesized.

## 1. Introduction

One way applied for the synthesis of substitute heteroaryl compounds is electrophilic aromatic substitution reactions. Due to their electron density, these reactions are faster with five-membered heteroaryl compounds than with benzene derivatives. Among furan, pyrrole, and thiophene, thiophene is more stable in the presence of protic acids or some reagents such as AlCl_3_ for electrophilic substitution [1].

Compounds containing the thiophene unit exhibit significant biological activity such as antimicrobial, antitumor, anti-inflammatory, and anticancer activities (Figure) [2–4]. Tiagabine (**1**), trade name *Gabitril*, is a drug used in the treatment of epilepsy [5]. Compound **2**, which is known, contains two carbonyl groups in addition to two thiophene units, like compound **1**. The synthesis of compound **2** was reported by different methods [6–9].

We synthesized some molecules in **3** structures, similar to compound **2**, and investigated their biological properties such as antioxidant activity [10–13]. The synthesis of compound **2** and an analogous compound from thiophene and related acyl chlorides can be considered. These compounds and their reactions may be interesting. These reactions may be bromination and reduction reactions in addition to reactions in the presence of a protic acid or Lewis acid such as AlCl_3_. Moreover, the biological activities of the products that may occur in these reactions can also be considered. Therefore, synthesis of the known compound **2** and the novel 1,7-di(thiophen-2-yl)heptane-1,7-dione (**4**) was carried out and their reactions were investigated.

## 2. Experimental section

### 2.1. General procedures

All commercial chemicals and solvents (some of them after purification) are suitable for all procedures. Values [melting point (Mp.), HRMS, IR, and NMR (Varian, 400 MHz in spectrometers)] written for the physical properties belonging to all compounds (whose some are known) are values obtained as previously reported [11,14].

### 2.2. Synthesis of 1,6-di(thiophen-2-yl)hexane-1,6-dione (2): Standard procedure for acylation

After adipoyl chloride (**6**) (1.08 g, 5.93 mmol) and AlCl_3_ (800 mg, 6.1 mmol) adding to a stirred solution of thiophene (**5**) (1.08 g, 12.86 mol) in CH_2_Cl_2_ (25 mL) at RT, the mixture was stirred at RT for 105 min. When the reaction was monitored with TLC (thin layer chromatography), it was observed that the reaction finished. After mixture was formed by adding H_2_O (25 mL) it was extracted with CH_2_Cl_2_ (3 × 50 mL), and combined organic phases were dried over Na_2_SO_4_ and filtered. CH_2_Cl_2_ was removed by rotary evaporator, crude was crystallized from EtOAc and compound **2** (1.45 g, 88%, white needle crystal) was obtained. Mp: 124–126 °C. (Lit. [8].: 125–126.5 °C); ^1^H-NMR (400 MHz, CDCl_3_): 7.72 (d, 2H, *J* = 3.60 Hz, aromatic), 7.63 (d, 2H, *J* = 4.89 Hz, aromatic), 7.12 (dd, 2H, *J* = 4.89, 3.60 Hz, aromatic), 2.96 (t, 4H, *J* = 6.60 Hz, methylenic), 1.87–1.82 (m, 4H, methylenic); ^13^C-NMR (100 MHz, CDCl_3_): 193.14 (CO), 144.53 (C), 133.74 (CH), 132.03 (CH), 128.32 (CH), 39.31 (CH_2_), 24.44 (CH_2_); HRMS: *m:z* (M+H)^+^ C_14_H_15_O_2_S_2_; for: 279.05135; found: 279.05338.

### 2.3. Synthesis of 1,7-di(thiophen-2-yl)heptane-1,7-dione (4)

According to the standard procedure written to obtain **2**, pimeloyl chloride (**7**) (2.35 g, 11.9 mmol) and AlCl_3_ (4.77 g, 36 mmol.), thiophene (**5**) (2.0 g, 23.7 mmol) and H_2_O (40 mL) were used. Time of the reaction (at RT) is 3 h. The product **4** (3.12 g, 89%) was obtained as brown crystal. Mp: 71–72 °C; ^1^H-NMR (400 MHz, CDCl_3_): 7.71 (dd, 2H, *J* = 3.8, 1.0 Hz, aromatic), 7.62 (dd, 2H *J* = 5.0, 1.0 Hz, aromatic), 7.12 (dd, 2H, *J* = 5.0, 3.8 Hz, aromatic), 2.92 (t, 2H, *J* = 7.4 Hz, methylenic), 1.84–1.76 (m, 4H, methylenic), 1.53–1.44 (m, 2H, methylenic); ^13^C-NMR (100 MHz, CDCl_3_): 193.24 (CO), 144.40 (C), 133.45 (CH), 131.78 (CH), 128.10 (CH), 39.06 (CH_2_), 28.88 (CH_2_), 24.37 (CH_2_); IR (CH_2_Cl_2_, *v*_max_, cm^−1^): 3090, 2937, 2863, 1656, 1519, 1416, 1355, 1234, 1056, 856; HRMS: *m:z* (M+H)^+^ C_15_H_16_O_2_S_2_; for: 293.067; found: 293.06669.

### 2.4. Synthesis of 1,6-di(1H-pyrrol-2-yl)hexane-1,6-dione (10)

According to the standard procedure written to obtain **2**, pyrrole (**8**) (1.1 g, 16.4 mmol), adipoyl chloride (**6**) (1.36 g, 7.5 mmol), AlCl_3_ (1.0 g, 7.5 mmol) and H_2_O (40 ml) were used. Time of the reaction (at RT) is 1.5 h. The product **10** (730 mg, 40%) was obtained from EtOAc as red amorphous. Mp: 128–130 °C; ^1^H-NMR (400 MHz, CDCl_3_): 9.80–9.53 (m, 2H, NH), 7.05–7.02 (m, 2H, aromatic), 6.94–6.53 (m, 2H, aromatic), 6.29–6.25 (m, 2H, aromatic), 2.81 (t, 4H, *J* = 5.12 Hz, methylenic), 1.83–1.78 (m, 4H, methylenic); ^13^C-NMR (100 MHz, CDCl_3_): 190.65 (CO), 131.95 (C), 124.66 (CH), 116.32 (CH), 110.64 (CH), 37.66 (CH_2_), 24.89 (CH_2_).

### 2.5. Synthesis of 1,6-di(furan-2-yl)hexane-1,6-dione (11)

According to the standard procedure written to obtain **2**, furan (**9**) (2.16 g, 31.8 mmol), adipoyl chloride (**6**) (2.66 g, 14.6 mmol), AlCl_3_ (1.96 g, 14.7 mmol) and H_2_O (50 mL) were used. Time of the reaction (at RT) is 25 min. Purification of crude on silica gel (55 g) column chromatography by EtOAc:hexane (1:9) elution gave product **11** (0.79 g, 22%, White crystal). Mp: 129–131 °C (Lit. [8].: 128–129.5 °C); ^1^H-NMR (400 MHz, CDCl_3_): 7.57 (dd, 2H, *J* = 1.59, 0.66 Hz, aromatic), 7.18 (dd, 2H, *J* = 3.53, 0.66 Hz, aromatic), 6.52 (dd, 2H, *J* = 3.53, 1.65 Hz, aromatic), 2.90–2.83 (m, 4H, methylenic), 1.84–1.76 (m, 4H, methylenic); ^13^C-NMR (100 MHz, CDCl_3_): 189.41 (CO), 152.92 (C), 146.50 (CH), 117.20 (CH), 112.41 (CH), 38.38 (CH_2_), 24.02 (CH_2_); HRMS: *m:z* (M+H)^+^ C_14_H_14_O_2_; for: 247.09703; found: 247.09651.

### 2.6. Synthesis of 1,6-di(thiophen-2-yl)hexane-1,6-diol (12): Standard procedure for reduction with NaBH_4_

NaBH_4_ (250 mg, 6.58 mmol) was carefully added to a stirred cold (at 0 °C) solution of the diketone **2** (250 mg, 0.9 mmol) in MeOH (25 mL) for 5 min. After the mixture at the same temperature for 10 min and the cold bath removed, it was stirred at RT for an addition day, then MeOH was evaporated by rotary evaporator, and water (15 mL) and EtOAc (40 mL) were added to residue. Separation of organic phase, extraction of aqueous phase with EtOAc (2 × 30 mL), and combination of organic phases were performed. Drying over Na_2_SO_4_ and filtration of the solution, and then removal of the EtOAc was also performed. Crystallization from EtOAc of crude gave diol **12** (240 mg, 95%, white crystals). Mp: 114–116 °C (Lit.[15]: 114.7–115.3 °C); ^1^H-NMR (400 MHz, CDCl_3_): 7.26–7.22 (m, 2H, aromatic), 6.97–6.94 (m, 4H, aromatic), 4.90 (dd, 2H, CHOH, *J* = 7.34, 6.05 Hz), 2.07 (s, 2H, OH), 1.95–1.74 (m, 4H, methylenic), 1.57–1.29 (m, 4H, methylenic); ^13^C-NMR (100 MHz, CDCl_3_): 148.99 (C), 126.82 (CH), 124.74 (CH), 123.94 (CH), 70.40 (CHOH), 39.32 (CH_2_), 25.71 (CH_2_).

### 2.7. Synthesis of 2,5-dibromo-1,6-di(thiophen-2-yl)hexane-1,6-dione (13): Standard procedure for bromination

To a stirred solution of **2** (250 mg, in CH_2_Cl_2_ (25 mL) was carefully added a mixture of bromine (486 mg) and CH_2_Cl_2_ (8 mL) at RT over 5 min. The volatile components of the mixture, which was stirred for another half hour, were evaporated. The residue was crystallized to give dibromide **13** (320 mg, 82%, pale yellow crystals) from EtOAc. Mp: 124–126 °C; ^1^H-NMR (400 MHz, CDCl_3_): 7.84 (d, 2H, *J* = 3.80 Hz, aromatic), 7.73 (d, 2H, *J* = 4.90 Hz, aromatic), 7.18 (dd, 2H, *J* = 4.90, 3.80 Hz, aromatic), 5.07–5.02 (m, 2H, CHBr), 2.54–2.43 (m, 2H, methylenic), 2.27–2.15 (m, 2H, methylenic); ^13^C-NMR (100 MHz, CDCl_3_): 186.00 (CO), 141.02 (C), 135.31 (CH), 133.28 (CH), 128.44 (CH), 46.81 (CHBr), 31.69 (CH_2_); IR (CH_2_Cl_2_, *v*_max_, cm^−1^): 3087, 3003, 2918, 2846, 1734, 1660, 1516, 1412, 1268, 1178, 1066, 854, 770, 719; HRMS: *m:z* (M+H)^+^ C_14_H_13_^79^Br^81^BrO_4_S_2_; for: 436.87032; found: 436.87468.

### 2.8. Reaction of 2 in the mixture of HCl/HOAc: Standard procedure for cyclocondensation

After addition of HCl (37%, 6 mL) at RT to a stirred solution of **2** (1.2 g, 4.3 mol) in HOAc (20 mL), the formed mixture was continued to be stirred for an additional 1 h at RT. Check with TLC showed that the reaction was complemented. After quenching by NaHCO_3_ solution (saturated, 40 mL) and its extraction with CH_2_Cl_2_ (2 × 50 mL), combined the organic phases were dried over Na_2_SO_4_, filtered, and its solvent was evaporated. The residue was submitted on silica gel column chromatography (60 g) with EtOAc:hexane (1:19). Respectively, the products **14** (720 mg, 64%, yellow crystal) and **15** (viscous, 225 g, 20%) were obtained from this purification.

#### Thiophen-2-yl(2-(thiophen-2-yl)cyclopent-1-en-1-yl)methanone (14)

Mp: 43–45 °C*;* ^1^H-NMR (400 MHz, CDCl_3_): 7.61 (dd, 1H, *J* = 4.92, 1.14 Hz, aromatic), 7.59 (dd, 1H, *J* = 3.79, 1.17 Hz, aromatic), 7.16 (dd, 1H, *J* = 5.09, 1.02 Hz, aromatic), 7.03–7.01 (m, 1H, aromatic), 7.00 (d, 1H, *J* = 3.97 Hz, aromatic), 6.87 (dd, 1H, *J* = 3.68, 5.08 Hz, aromatic), 3.03–2.91 (m, 4H, methylenic), 2.16–2.06 (m, 2H, methylenic); ^13^C-NMR (100 MHz, CDCl_3_): 190.40 (CO), 144.03 (C), 138.36 (C), 137.43 (C), 136.23 (C), 134.74 (CH), 134.19 (CH), 128.40 (CH), 127.37 (CH), 127.29 (CH), 126.62 (CH), 38.30 (CH_2_), 38.27 (CH_2_), 23.04 (CH_2_); IR (CH_2_Cl_2_, *v*_max_, cm^−1^): 3101, 2953, 2845, 1626, 1514, 1428, 1411, 1353, 1272, 1248, 1229, 1133, 1058, 848, 786, 724, 703;. HRMS: *m:z* (M^+^) C_14_H_12_OS_2_; for: 260.0330; found: 260.0324.

#### Thiophen-2-yl(2-(thiophen-2-yl)cyclopent-2-en-1-yl)methanone (15)

^1^H-NMR (400 MHz, CDCl_3_): 7.86 (d, 1H, *J* = 3.70 Hz, aromatic), 7.66 (d, 1H, *J* = 5.00 Hz, aromatic), 7.15 (t, 1H, *J* = 4.30 Hz, aromatic), 7.09 (d, 1H, *J* = 5.10 Hz, aromatic), 6.84 (t, 1H, *J* = 4.40 Hz, aromatic), 6.75 (d, 1H, *J* = 3.40 Hz, aromatic), 4.70–4.63 (m, 1H, olefinic), 2.78–2.67 (m, 1H, methylenic), 2.65–2.56 (m, 2H, methylenic), 2.55–2.46 (m, 1H, methylenic), 2.25–2.16 (m, 1H, methylenic); ^13^C-NMR (100 MHz, CDCl_3_): 194.20 (CO), 143.73 (C), 139.65 (C), 135.51 (C), 134.00 (CH), 132.25 (CH), 130.16 (CH), 128.22 (CH), 127.26 (CH), 124.12 (CH), 123.97 (CH), 56.40 (CH), 32.55 (CH_2_), 30.39 (CH_2_). IR (CH_2_Cl_2_, *v*_max_, cm^−1^): 3103, 2920, 2849, 1745, 1655, 1516, 1355, 1324, 1236, 1212, 1140, 1082, 1063, 971, 860, 777, 728, 701; HRMS: *m:z* (M+H)^+^ C_14_H_13_OS_2_; for 261.04078; found: 261.04053.

### 2.9. Synthesis of (E)-N′-(thiophen-2-yl(2-(thiophen-2-yl)cyclopent-1-en-1-yl)methylene)acetohydrazide (16)

After hydrazine hydrate (250 mg, 7.81 mmol) was added to a stirred solution of **14** (300 mg, 1.15 mmol) in HOAc (20 mL) and the mixture was refluxed for 3 days, it was poured into mixture of ice-water (200 g). While neutralization of the mixture realized by a solution (concerted) of NH_3_, it was controlled with pH paper. One day later, it was observed that no precipitate formed in the mixture. After extraction with CH_2_Cl_2_ (3 × 70 mL) of the mixture and drying over Na_2_SO_4_ of combined the organic phases, it was filtered and CH_2_Cl_2_ was removed. Crystallization of crude with EtOAc gave product **16** (125 mg, 34%, white crystals). Mp: 156–158 °C; ^1^H-NMR (400 MHz, CDCl_3_): 8.49 (s, 1H, NH), 7.34 (d, 1H, *J* = 5.02 Hz, aromatic), 7.19 (d, 1H, *J* = 5.02 Hz, aromatic), 7.10 (d, 1H, *J* = 3.55 Hz, aromatic), 7.02 (d, 1H, *J* = 3.41 Hz, aromatic), 6.99–6.95 (m, 1H, aromatic), 6.94–6.91 (m, 1H, aromatic), 3.08–3.00 (m, 2H, methylenic), 2.82–2.67 (m, 2H, methylenic), 2.37 (s, 3H, methyl), 2.22–2.13 (m, 2H, methylenic); ^13^C-NMR (100 MHz, CDCl_3_): 172.87 (CO), 145.24 (C), 140.23 (C), 138.44 (C), 136.98 C), 127.93 (CH), 127.80 (CH), 127.47 (CH), 127.31 (CH), 127.10 (CH), 126.50 (CH), 125.80 (C), 36.94 (CH_2_), 36.70 (CH_2_), 22.56 (CH_2_), 20.50 (CH_3_); IR (CH_2_Cl_2_, *v*_max_, cm^−1^): 3735, 3303, 3167, 3074, 2913, 2844, 1673, 1567, 1525, 1451, 1429, 1381, 1326, 1297, 1276, 1230, 1152, 1120, 1045, 1014, 908, 873, 852, 831, 802, 736, 701. HRMS: *m:z* (M^+^) C_16_H_16_N_2_OS_2_; for: 316.0704; found: 316.0699.

### 2.10. Synthesis of thiophen-2-yl(2-(thiophen-2-yl)cyclopentyl)methanone (17)

Pd/C catalyst (10 mg), the compound **14** (370 mg, 1.42 mmol) and MeOH (25 mL) were placed in the flask (100 mL, two necked, round-bottomed) provided with a spinbar at RT. The gas that was first air in the flask was replaced 3 times with hydrogen gas, which is in the balloon attached to the flask and about 1 atm, and then the **14** was reacted with hydrogen gas for 6 days. Filtering the reaction mixture through a filter paper to remove the catalyst followed by evaporation of MeOH gave the compound **17** (345 mg, 93%, yellow liquid) as the sole product. ^1^H-NMR (400 MHz, CDCl_3_): 7.54 (d, 1H, *J* = 3.8 Hz, aromatic), 7.50 (d, 1H, *J* = 4.7 Hz, aromatic), 7.00 (dd, 1H, *J* = 4.8, 4.0 Hz, aromatic), 6.94 (dd, 1H, *J* = 5.0, 0.8 Hz, aromatic), 6.73 (dd, 1H, *J* = 5.0, 3.6 Hz, aromatic), 6.66 (d, 1H, *J* = 3.4 H*z*, aromatic), 3.95 (td, 1H, *J* = 7.8, 6.0 Hz, methylenic), 3.81 (dd, 1H, *J* = 16.2, 8.1 Hz, methylenic), 2.35–2.25 (m, 1H, methylenic), 2.25–2.16 (m, 2H, methylenic), 2.15–2.05 (m, 1H, methylenic), 2.04–1.94 (m, 1H, methylenic), 1.86–1.72 (m, 1H, methylenic); ^13^C-NMR (100 MHz, CDCl_3_): 194.63 (CO), 145.51 (C), 144.37 (C), 133.20 (CH), 131.54 (CH), 127.73 (CH), 126.40 (CH), 124.73 (CH), 123.08 (CH), 53.07 (CH), 45.73 (CH), 33.82 (CH_2_), 28.37 (CH_2_), 24.04 (CH_2_); IR (CH_2_Cl_2_, *v*_max_, cm^−1^): 3102, 2954, 2869, 1657, 1518, 1438, 1416, 1362, 1305, 1266, 1237, 1080, 1059, 1038, 839, 780, 722, 695, 526; HRMS: *m:z* (M^+^) C_14_H_14_OS_2_; for: 262.0486; found: 262.0481.

### 2.11. Bromination of the compound 4

According to the standard procedure written for bromination of **2**, the compound **4** (180 mg, 0.62 mmol), Br_2_ (0.5 mL), AlCl_3_ (1.0 g, 7.5 mmol), and H_2_O (40 mL) were used. Time of the reaction (at RT) is 5 h. Purification of crude on the preparative thick-layer chromatography (PTkLC) using EtOAc/hexane (1:9) gave monobromide **18** (95 mg, 42%, viscose) and dibromide **19** (135 mg, 49%, viscose) were obtained, respectively.

#### 2-Bromo-1,7-di(thiophen-2-yl)heptane-1,7-dione (18)

^1^H-NMR (400 MHz, CDCl_3_): ^1^H NMR (400 MHz, CDCl_3_): 8.32 (dd, *J* = 4.0, 0.9 Hz, 1H, aromatic), 7.85 (dd, 1H, *J* = 3.9, 1.0 Hz, aromatic), 7.72 (dd, 1H, *J* = 5.0, 0.9 Hz, aromatic), 7.68 (dd, 1H, *J* = 5.0, 1.1 Hz, aromatic), 7.20–7.14 (m, 2H, aromatic), 5.03 (dd, 1H, CHBr, *J* = 7.9, 6.4 Hz), 2.80–2.65 (m, 2H, methylenic), 2.41–2.20 (m, 2H, methylenic), 2.11–1.86 (m, 2H, methylenic), 1.35–1.19 (m, 1H, methylenic), 0.95–0.82 (m, 1H, methylenic); ^13^C NMR (101 MHz, CDCl_3_): 186.27 (CO), 182.12 (CO), 141.20 (C), 137.72 (C), 136.34 (CH), 135.14 (CH), 135.11 (CH), 133.15 (CH), 128.37 (CH), 127.88 (CH), 64.95 (CHBr), 47.40 (CH_2_), 45.62 (CH_2_), 32.86 (CH_2_), 25.64 (CH_2_); IR (CH_2_Cl_2_, *v*_max_, cm^−1^): 2921, 1655, 1410, 1251, 1060, 750. HRMS: *m:z* (M-HBr+3H)^+^ C_15_H_17_O_2_S_2_; for 293.06700; found: 293.06650.

#### 2,6-Dibromo-1,7-di(thiophen-2-yl)heptane-1,7-dione (19)

^1^H-NMR (400 MHz, CDCl_3_): 7.85–7.81 (m, 2H, aromatic), 7.74–7.69 (m, 2H, aromatic), 7.20–7.15 (m, 2H, aromatic), 4.98 (t, 2H, *J* = 7.2 Hz, CHBr), 2.37–2.09 (m, 4H, methylenic), 1.90–1.61 (m, 2H, methylenic); ^13^C NMR (100 MHz, CDCl_3_): 186.23 (CO), 141.28 (C), 135.38 (CH), 128.46 (CH), 47.43 (CH_2_), 47.23 (CH_2_), 33.01 (CH_2_), 32.97 (CH_2_), 25.47 (CH_2_); IR (CH_2_Cl_2_, *v*_max_, cm^−1^): 2923, 1660, 1516, 1412, 1354, 1258, 1059, 858, 723. HRMS: m:z (M+H)^+^ C_15_H_14_^79^Br_2_O_2_S_2_; for: 448.88802; found: 447.8832.

### 2.12. Synthesis of 1,7-di(thiophen-2-yl)heptane-1,7-diol (20)

According to the standard procedure written to obtain **12**, NaBH_4_ (0.25 g, 6.58 mmol) and the compound **4** (130 mg, 0.45 mmol) were used. The time is 2.5 d at RT. Diol **20** (60 mg, 46%) was obtained as transparent liquid. ^1^H-NMR (400 MHz, CDCl_3_): 7.24–7.20 (m, 2H, aromatic), 6.96–6.92 (m, 4H, aromatic), 4.90–4.83 (m, 2H, CHO), 2.24 (s, 2H, OH), 1.91–1.71 (m, 4H, methylenic), 1.50–1.23 (m, 6H, methylenic); ^13^C NMR (101 MHz, CDCl_3_): 148.89 (C), 126.59 (CH), 124.46 (CH), 123.70 (CH), 70.25 (CHO), 39.14 (CH_2_), 29.07 (CH_2_), 25.62 (CH_2_). IR (CH_2_Cl_2_, *v*_max_, cm^−1^): 3361, 2932, 2857, 1276, 1170, 1035, 832, 698; HRMS: m:z (M-H_2_O)^+^ C_15_H_19_OS_2_; for: 279.08773; found: 279.08799.

### 2.13. Reaction of diketone 4 in the mixture of HCl/HOAc

According to the standard procedure written to obtain **15** and **16**, the diketone **4** (200 mg, 0.68 mmol), H_2_O (40 mL) and HCl (37%, 4 mL) were used. The time of the reaction (at RT) is 2 h. Purification of crude on the preparative thick-layer chromatography (PTkLC) using EtOAc/hexane (1:9) gave the product **21** (110 mg, 59%, dark red viscose) and the product **22** (60 mg, 32%, dark red viscose), respectively.

#### Thiophen-2-yl(2-(thiophen-2-yl)cyclohex-1-en-1-yl)methanone (21)

^1^H-NMR (400 MHz, CDCl_3_) 7.51 (d, 1H, *J* = 4.8 Hz, aromatic), 7.45 (dd, 1H, *J* = 3.4, 0.6 Hz, aromatic), 7.10 (d, 1H, *J* = 5.2 Hz, aromatic), 6.95–6.92 (m, 1H, aromatic), 6.88 (d, 1H, *J* = 3.5 Hz, aromatic), 6.76 (dd, 1H, *J* = 5.0, 3.7 Hz, aromatic), 2.56 (td, 2H, *J* = 6.0, 2.9 Hz, methylenic), 2.47 (td, 2H, *J* = 6.0, 2.9 Hz, methylenic), 1.91–1.83 (m, 2H, methylenic), 1.83–1.76 (m, 2H, methylenic); ^13^C NMR (100 MHz, CDCl_3_): 193.57 (CO), 143.73 (2C), 135.99 (C), 133.99 (CH), 133.50 (CH), 131.16 (C), 127.79 (CH), 126.94 (CH), 126.41 (CH), 125.26 (CH), 31.27 (CH_2_), 28.47 (CH_2_), 22.74 (CH_2_), 21.86 (CH_2_); IR (CH_2_Cl_2_, *v*_max_, cm^−1^): 3521, 2922, 1633, 1410, 1261, 859, 905, 752, 520; HRMS: m:z (M+H)^+^ C_15_H_15_OS_2_; for: 275.05643; found: 275.05588.

#### Thiophen-2-yl(2-(thiophen-2-yl)cyclohex-2-en-1-yl)methanone (22)

^1^H-NMR (400 MHz, CDCl_3_): 7.86 (dd, 1H, *J* = 1.1, 3.6 Hz, aromatic), 7.65 (dd, 1H, *J* = 5.0, 0.8 Hz, aromatic), 7.16 (dd, 1H, *J* = 4.8, 3.9 Hz, aromatic), 7.02 (dd, 1H, *J* = 5.0, 0.6 Hz, aromatic), 6.82 (dd, 1H, *J* = 5.1, 3.7 Hz, aromatic), 6.76 (d, 1H, *J* = 3.4 Hz, aromatic), 6.49 (t, 1H, *J* = 4.1 Hz, aromatic), 4.42 (t, 1H, *J* = 4.6 Hz, olefinic), 2.40–2.18 (m, 2H, methylenic), 2.15–2.04 (m, 2H, methylenic), 1.82–1.60 (m, 2H, methylenic); ^13^C NMR (100 MHz, CDCl_3_): 193.67 (CO), 145.78 (C), 143.37 (C), 133.74 (CH), 131.91 (CH), 128.89 (C), 128.25 (CH), 128.21 (CH), 127.17 (CH), 122.99 (CH), 121.60 (CH), 47.36 (CH), 27.60 (CH_2_), 25.35 (CH_2_), 18.35 (CH_2_). IR (CH_2_Cl_2_, *v*_max_, cm^,−1^): 2924, 1657, 1413, 1261, 748; HRMS: m:z (M+H)^+^ C_15_H_15_OS_2_; for: 275.05643; found: 275.05588.

## 3. Result and discussion

**Scheme 1.**
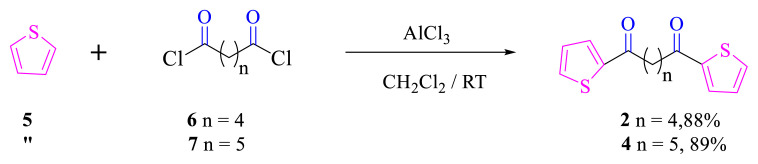
The synthesis of compounds **2** and **4**.

Based on a method in the literature [6], each of compounds **2** and **4** was obtained as a result of the reactions of thiophene with the corresponding diacyl chlorides (adipoyl chloride or pimeloyl chloride) (Scheme 1).

**Scheme 2.**
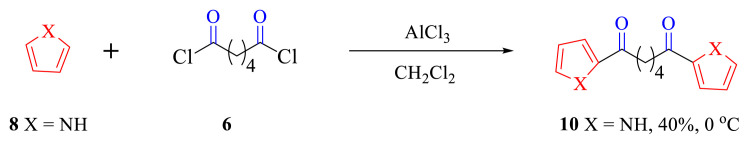
The synthesis of compounds **10** and **11** in the presence of AlCl_3_.

Compounds with pyrrole and furan units in place of thiophene units in compound **2** are known. However, to the best of our knowledge, these compounds **10** and **11** were not synthesized in the presence of AlCl_3_ [7–9,16]. Each of these compounds was also synthesized in the presence of AlCl_3_ (Scheme 2).

**Scheme 3.**
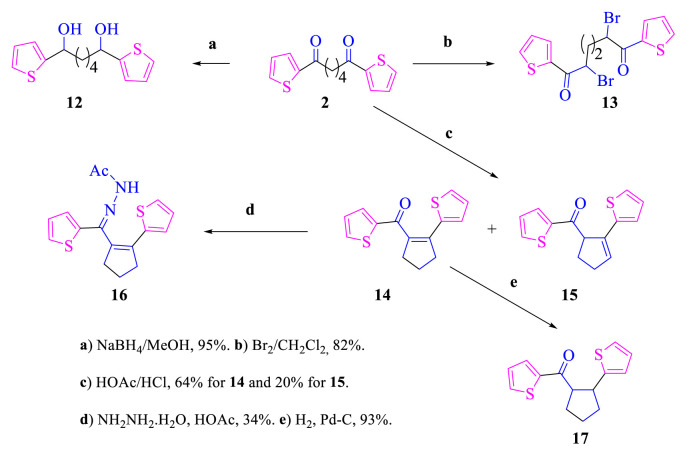
The reactions of compounds **2**.

Reduction of diketone **2** with NaBH_4_ was performed because benzylic ketones reduce NaBH_4_ [10,11,17]. Diol **12** synthesized by another method [15] was obtained in high yield from this reaction. Molecular bromine reacts with electron-rich aromatic rings and α-hydrogens of ketones [10,11,18–20]. To establish whether there was regioselectivity in the reaction of bromine with compound **2**, bromine was reacted with compound **2**. It was observed that dibromide **13** occurred regioselectively in this reaction (Scheme 3).

Some compounds are condensed (or rearranged) to give products under different conditions, and these products may be mechanistically significant. In fact, they may be a targeted compound or starter product for some compounds. 1,6-Diketones cyclocondensed (rearranged) to give compounds including a five-membered ring in acidic media [10,11,21–23]. To detect cyclocondensation (arrangement) in compound **2**, it was mixed with HOAc/HCl and monitored by thin-layer chromatography (TLC) at RT. After completion of this reaction and purification of the crude product by silica gel column chromatography, two products were obtained (Scheme 3). According to the NMR spectra of these products, all the carbons and hydrogens in the molecules were different and there was one carbonyl group in both molecules. In addition, one of the products has olefinic hydrogen (at 4.70–4.63 ppm as m and 1H). Thus, the products with and without olefinic hydrogen were identified as **15** and **14**, respectively. These products must be cyclocondensation or rearrangement products like known compounds in the literature [10,11,21,24].

Derivatives of compound **14** may be important because it is an α, β-unsaturated compound. Compound **14** was reacted with hydrazine hydrate in HOAc. The presence of a large number of products was observed in the reaction mixture according to monitoring with TLC and its ^1^H-NMR spectrum. Crystallization of the crude solid formed with EtOAc gave product **16**. Reduction product **17** was easily obtained from catalytic hydrogenation of compound **16**. Evidence for the structures of compounds **16** and **17** is the presence of a methyl peak at 2.37 ppm (s, 3H) in the ^1^H-NMR spectrum of **16** and five peaks in the aliphatic region of the ^13^C-NMR spectrum of **17**.

**Scheme 4.**
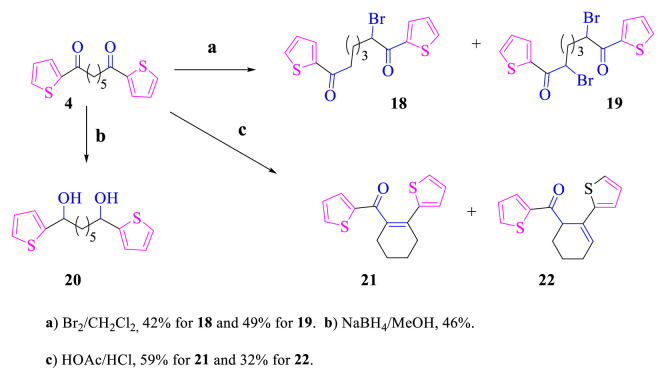
The reactions of compounds **4**.

Compound **4** is very similar to compound **2**. Therefore, most of its reactions have been performed like that of compound **2** (Scheme 4). Regioselective bromination of **4** with molecular bromine gave monobromide **18** and dibromide **19**. Likewise, diol **20** was also obtained from the reduction reaction of **4** with NaBH_4_. Compounds **21** and **22** including a six-membered ring were also synthesized from the reaction of compound **4** in HOAc/HCl.

**Scheme 5.**
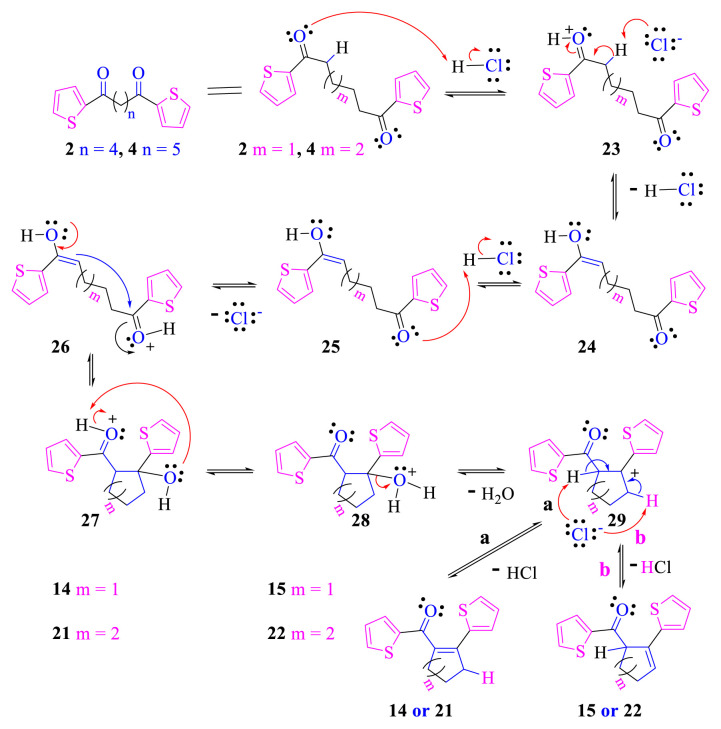
The formation mechanism of compounds **14**, **15**, **21** and **22**.

The reaction mechanism shown in Scheme 5 is proposed for the formation of cyclocondensation products **14**, **15**, **21**, and **22** from the corresponding compounds **2** or **4**. Intermediate **25**, in which a carbonyl group in **2** or **4** is converted to its enol form in acidic medium occurs via intermediates **23** and **24**. Intermediate **27** is formed when the enol group in intermediate **26** attacks the carbon of the protonated carbonyl group. By dehydration of water via **28**, carbocation **29** is formed. As shown in Scheme 5, two isomeric compounds containing double bonds at different positions can be obtained by attacks of Cl- ions on two different hydrogen atoms in carbocation **29** like ways **a** and **b**. Therefore, the products **14** or **21** and **15** or **22** are formed by ways **a** and **b**, respectively.

## 4. Conclusion

Compound **2**, which is known, and compound **4**, which is novel, each containing two CO and thiophene groups, were obtained from the reaction of thiophene with the corresponding adipoyl and pimeloyl chlorides in the presence of AlCl_3_, respectively. Synthesis in different ways of each of the known diketones **10** and **11**, containing pyrrole and furan rings in place of the thiophene rings in diketone **2**, was reported [7–9,16]. They were also synthesized similarly to how **2** was synthesized. This is an additional synthesis method for them.

Reactions such as bromination, condensation, and reductions of compounds **2** and **4** were carried out. Among these reactions, the cyclocondensation reactions are more important than others because the products **14**, **15**, **21**, and **22** were formed from reactions of compounds **2** or **4** in HOAc/HCl. The formations of the cyclocondensation products **14**, **15**, **21**, and **22** may also be described as rearrangement. Cyclocondensation products containing five or six-membered rings contain two thiophene units. To the best of our knowledge, cyclocondensation products containing five or six-membered rings, including two heteroaromatic rings such as thiophene, are unknown.

Molecular bromine with the α hydrogen of the carbonyl group in **2** and **4** gave the substitution reactions. Products with bromine in the thiophene rings were not obtained by bromination of **2** and **4**. As can be seen in Schemes 3 and 4, bromides **13**, **18**, and **19** were regioselectively obtained. While the thiophene ring reacts rapidly with bromine even at low temperatures (≤0 °C) [1], the reason why the thiophene rings in compounds **2** and **4** do not react with bromine is thought to be the carbonyl groups attached to the thiophene rings. Carbonyl groups reduce the electron density of thiophene rings because they are electron-withdrawing groups. While cyclopropane rings react with reagents such as Br_2_ and H_2_ (with Pd/C), cyclopropane rings attached to the ester group do not react with these reagents [25,26].

In the present work, four known compounds (**2** and **10–12**) and eleven novel compounds (**4**, **13– 22**) were synthesized. The purification and structure determination of all the compounds synthesized were achieved by various methods.

## Supplementary Material

### NMR spectra of synthesized compounds

^1^H-NMR spectrum of the compound **2** (400 MHz, CDCl_3_).

^13^C-NMR spectrum of the compound **2** (CDCl_3_, 100 MHz).

^1^H-NMR spectrum of the compound 4 (400 MHz, CDCl_3_).

^13^C-NMR spectrum of the compound **4** (CDCl_3_, 100 MHz).

^1^H-NMR spectrum of the compound **10** (400 MHz, CDCl_3_).

^13^C-NMR spectrum of the compound **10** (CDCl_3_, 100 MHz).

^1^H-NMR spectrum of the compound **11** (400 MHz, CDCl_3_).

^13^C-NMR spectrum of the compound **11** (CDCl_3_, 100 MHz).

^1^H-NMR spectrum of diol **12** (400 MHz, CDCl_3_).

^13^C-NMR spectrum of diol **12** (CDCl_3_, 100 MHz).

^13^C-NMR spectrum of dibromide **13** (CDCl_3_, 100 MHz).

^1^H-NMR spectrum of dibromide **13** (400 MHz, CDCl_3_).

^1^H-NMR spectrum of the compound **14** (400 MHz, CDCl_3_).

^13^C-NMR spectrum of the compound **14** (CDCl_3_, 100 MHz).

^1^H-NMR spectrum of the compound **15** (400 MHz, CDCl_3_).

^13^C-NMR spectrum of the compound **15** (CDCl_3_, 100 MHz).

^1^H-NMR spectrum of the compound **16** (400 MHz, CDCl_3_).

^13^C-NMR spectrum of the compound **16** (100 MHz, CDCl_3_).

^1^H-NMR spectrum of the compound **17** (400 MHz, CDCl_3_).

^13^C-NMR spectrum of the compound **17** (100 MHz, CDCl_3_).

^1^H-NMR spectrum of the compound **18** (400 MHz, CDCl_3_).

^13^C-NMR spectrum of the compound **18** (100 MHz, CDCl_3_).

^1^H-NMR spectrum of diromide **19** (400 MHz, CDCl_3_).

^13^C-NMR spectrum of diromide **19** (100 MHz, CDCl_3_).

^1^H-NMR spectrum of diol **20** (400 MHz, CDCl_3_).

^13^C-NMR spectrum of diol **20** (100 MHz, CDCl_3_).

^1^H-NMR spectrum of the compound **21** (400 MHz, CDCl_3_).

^13^C-NMR spectrum of the compound **21** (100 MHz, CDCl_3_).

^1^H-NMR spectrum of the compound **22** (400 MHz, CDCl_3_).

^13^C-NMR spectrum of the compound **22** (100 MHz, CDCl_3_).

## Figures and Tables

**Figure.**
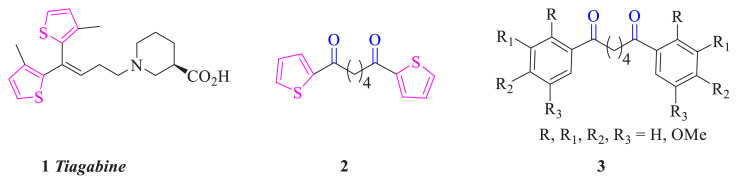
Some important compounds.
