# Supplementary figures and images for: Synthesis and reactions of di(thiophen-2-yl)alkane diones: Cyclocondensation
Source: Turk J Chem. 2022 Apr 28;46(5):1397–404. doi: 10.55730/1300-0527.3446 (PMC10390138; doi:10.55730/1300-0527.3446)

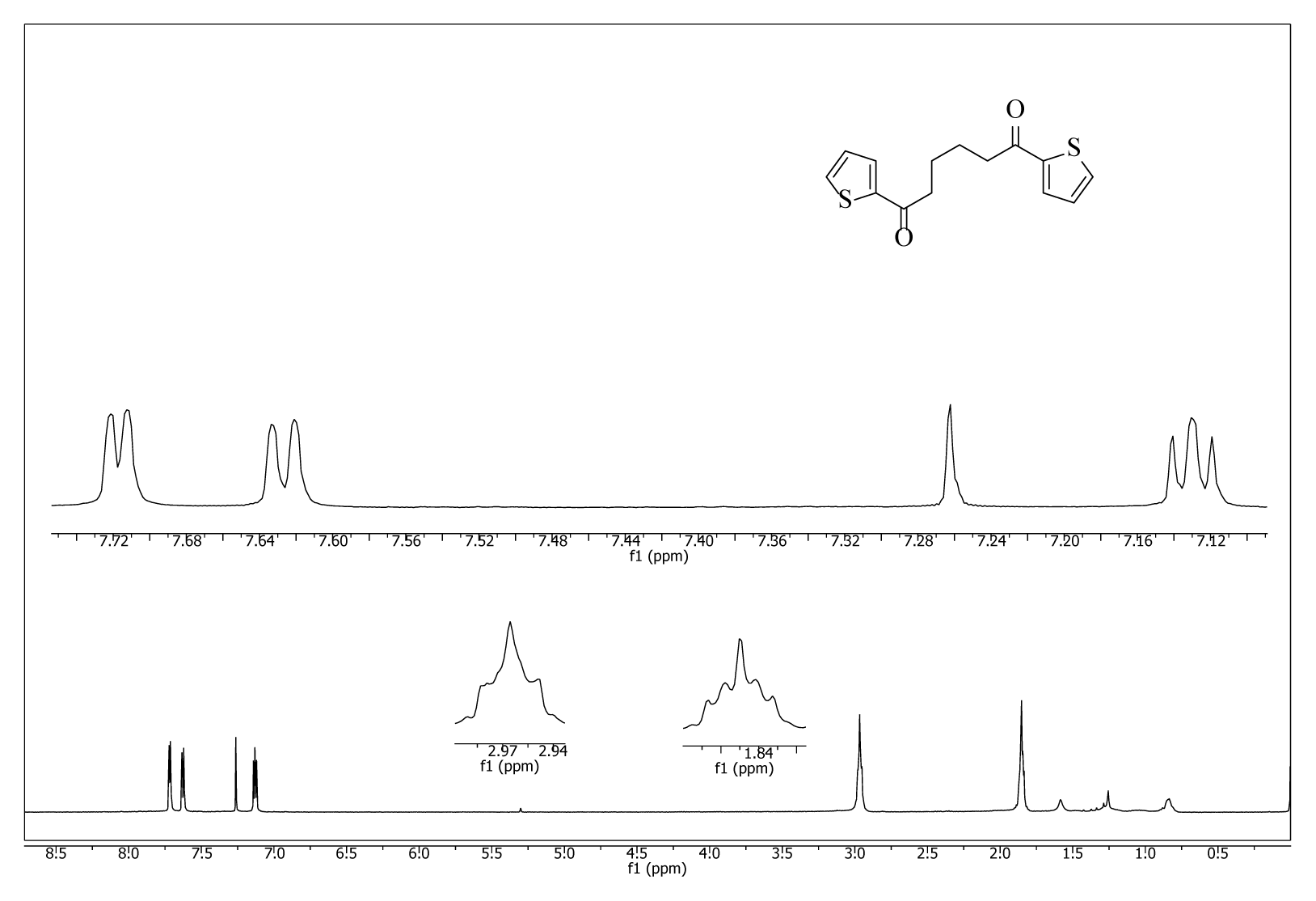

Supplement: Supplementary file 1 — 1H-NMR spectrum of the compound 2 (400 MHz, CDCl3). [file turkjchem-46-5-1397s1.tif]

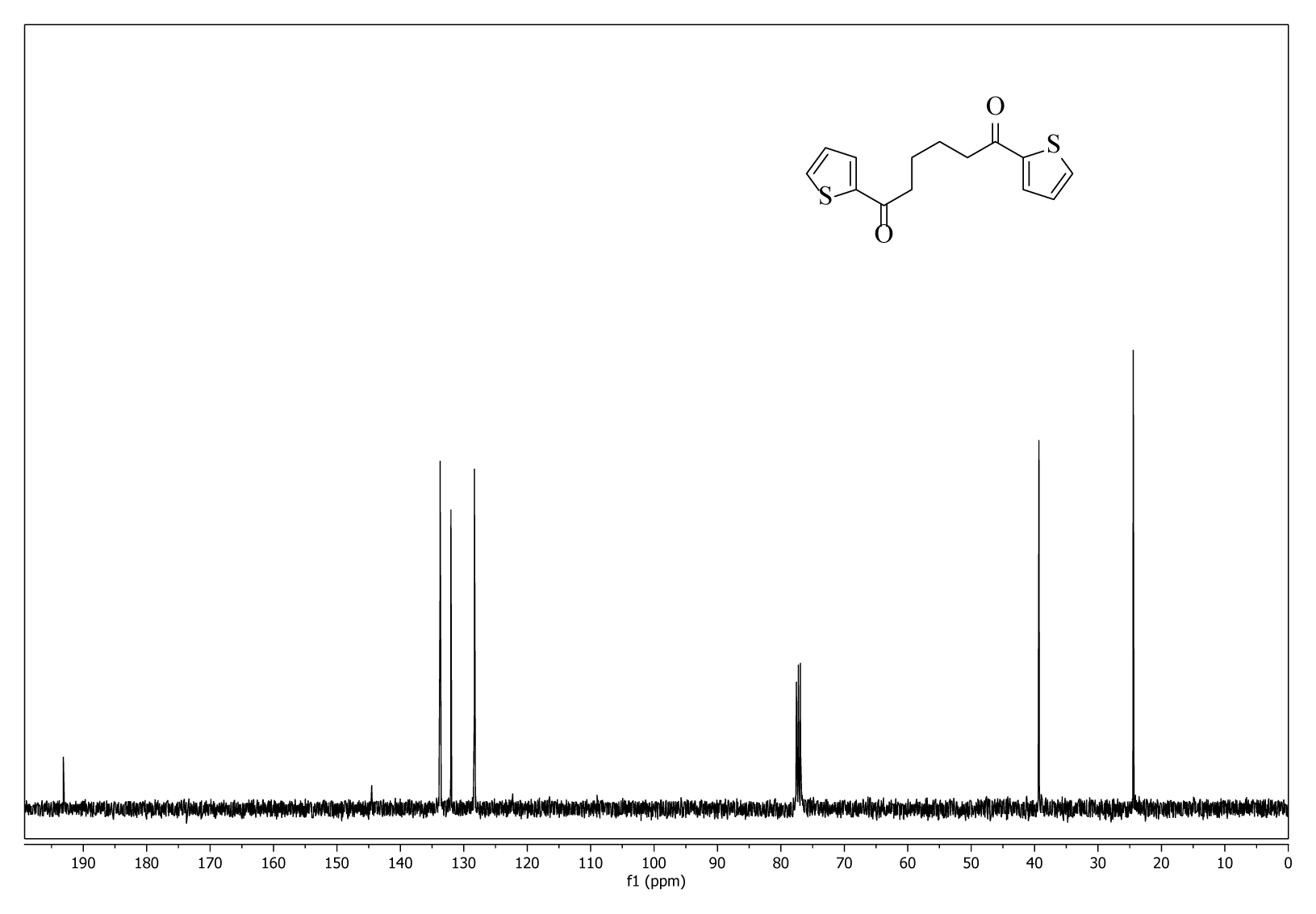

Supplement: Supplementary file 2 — 13C-NMR spectrum of the compound 2 (CDCl3, 100 MHz). [file turkjchem-46-5-1397s2.tif]

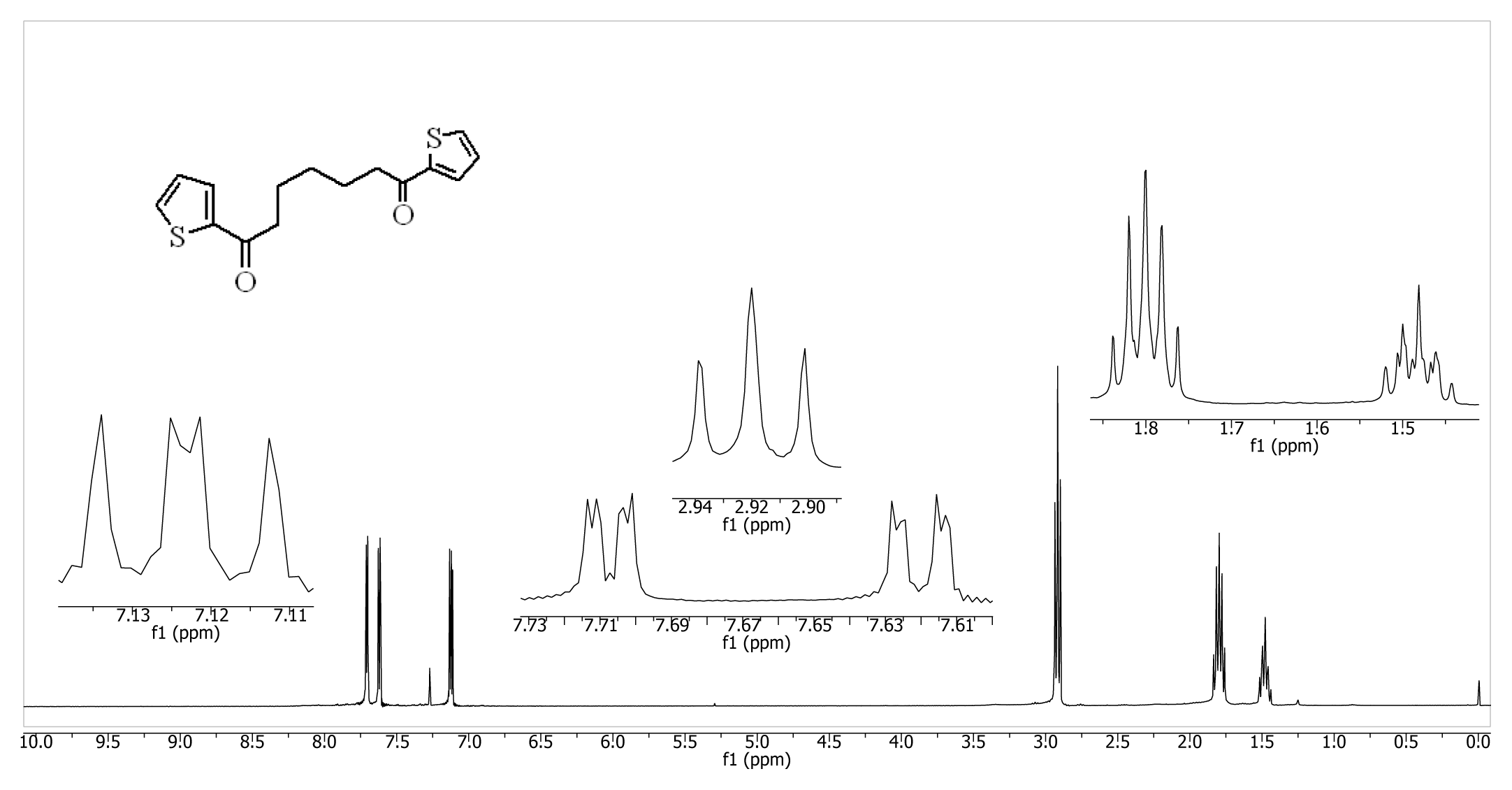

Supplement: Supplementary file 3 — 1H-NMR spectrum of the compound 4 (400 MHz, CDCl3). [file turkjchem-46-5-1397s3.tif]

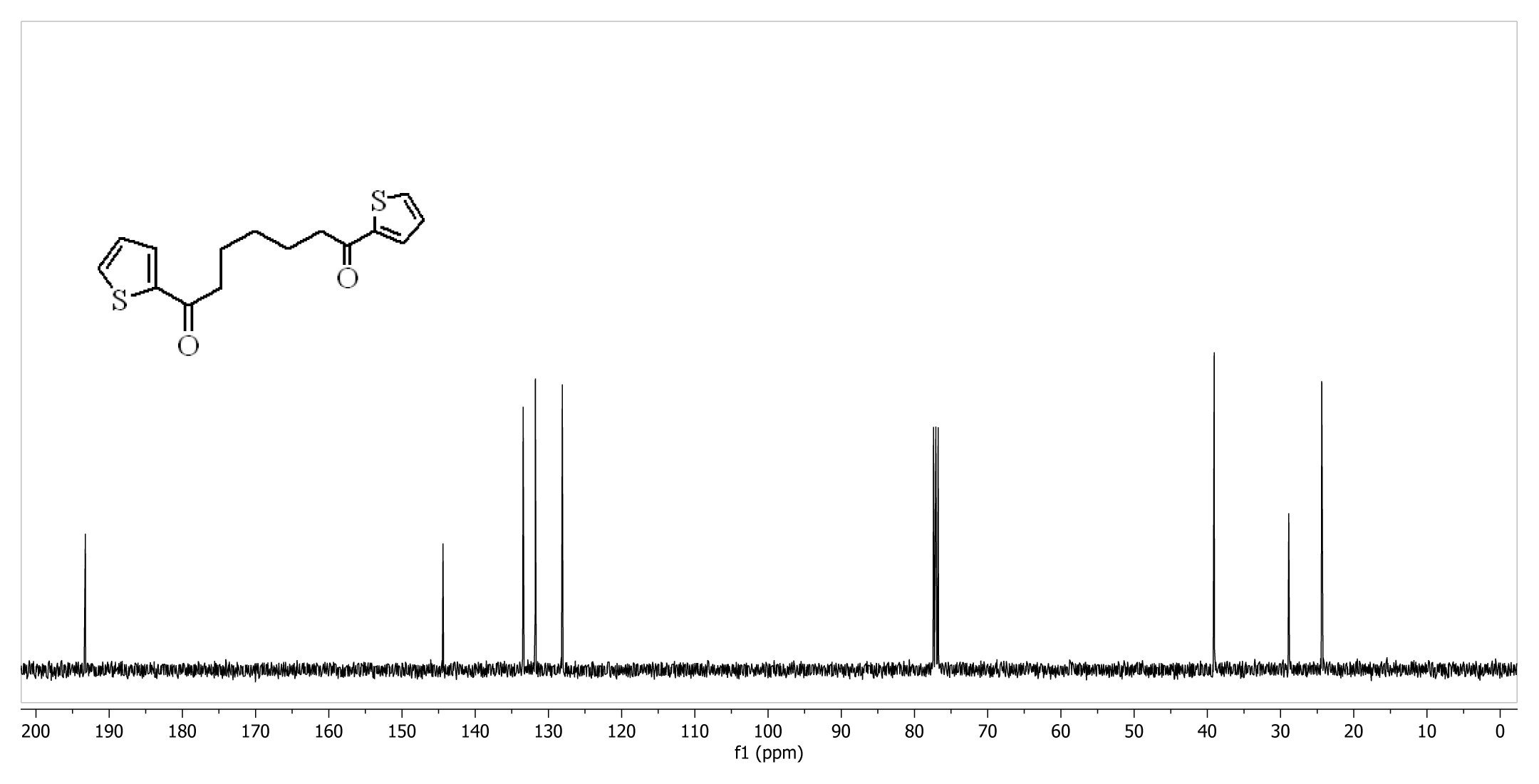

Supplement: Supplementary file 4 — 13C-NMR spectrum of the compound 4 (CDCl3, 100 MHz). [file turkjchem-46-5-1397s4.tif]

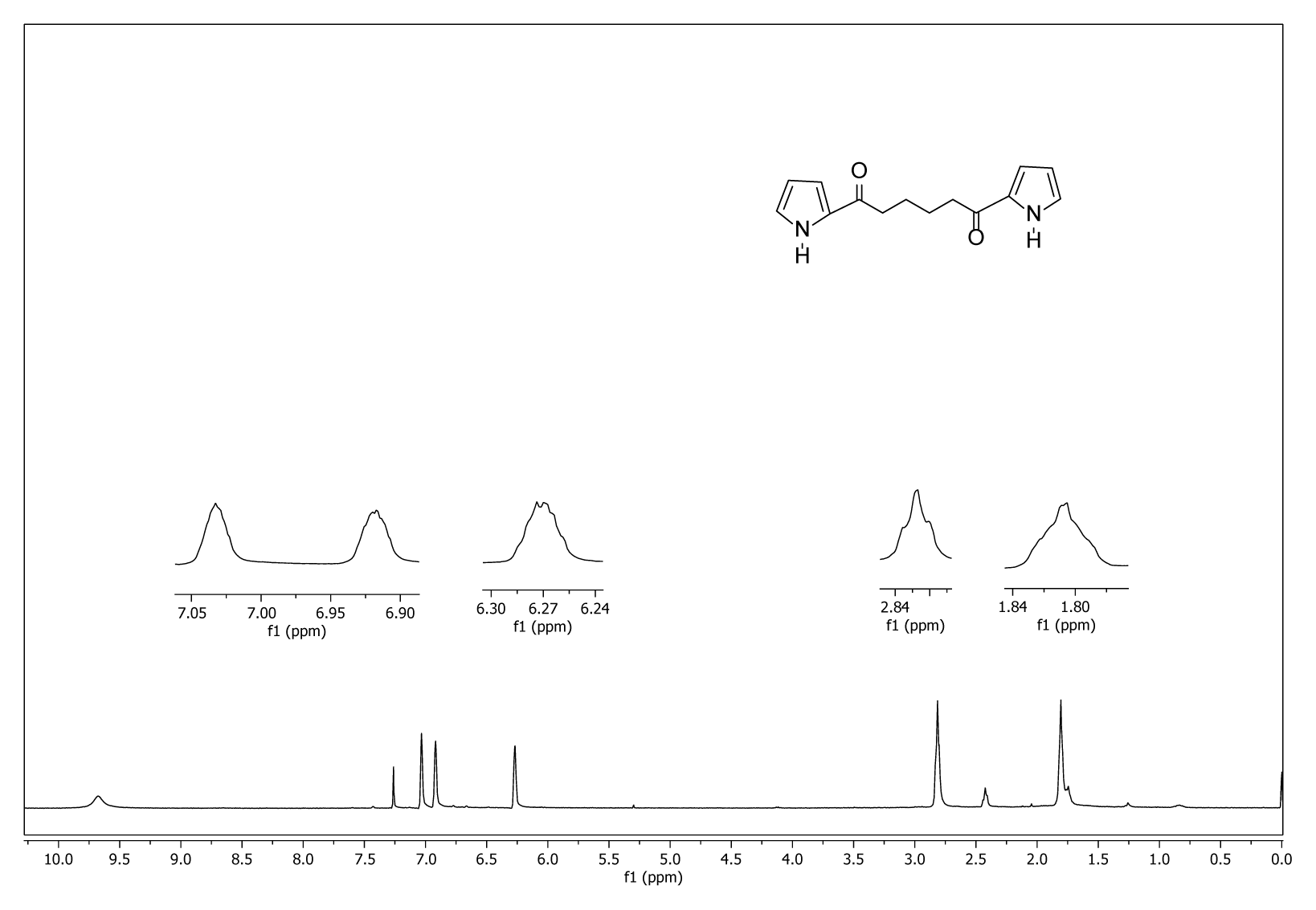

Supplement: Supplementary file 5 — 1H-NMR spectrum of the compound 10 (400 MHz, CDCl3). [file turkjchem-46-5-1397s5.tif]

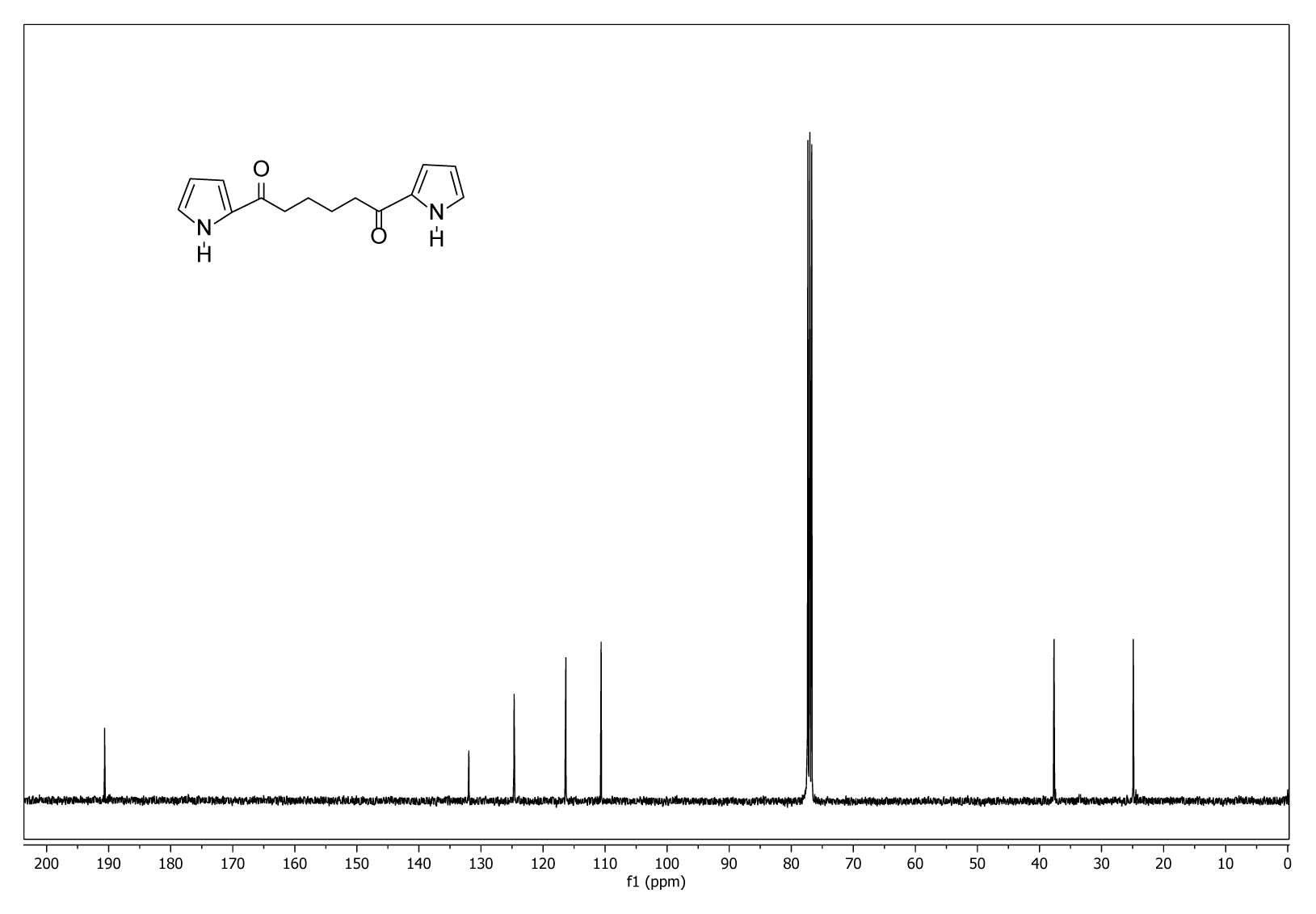

Supplement: Supplementary file 6 — 13C-NMR spectrum of the compound 10 (CDCl3, 100 MHz). [file turkjchem-46-5-1397s6.tif]

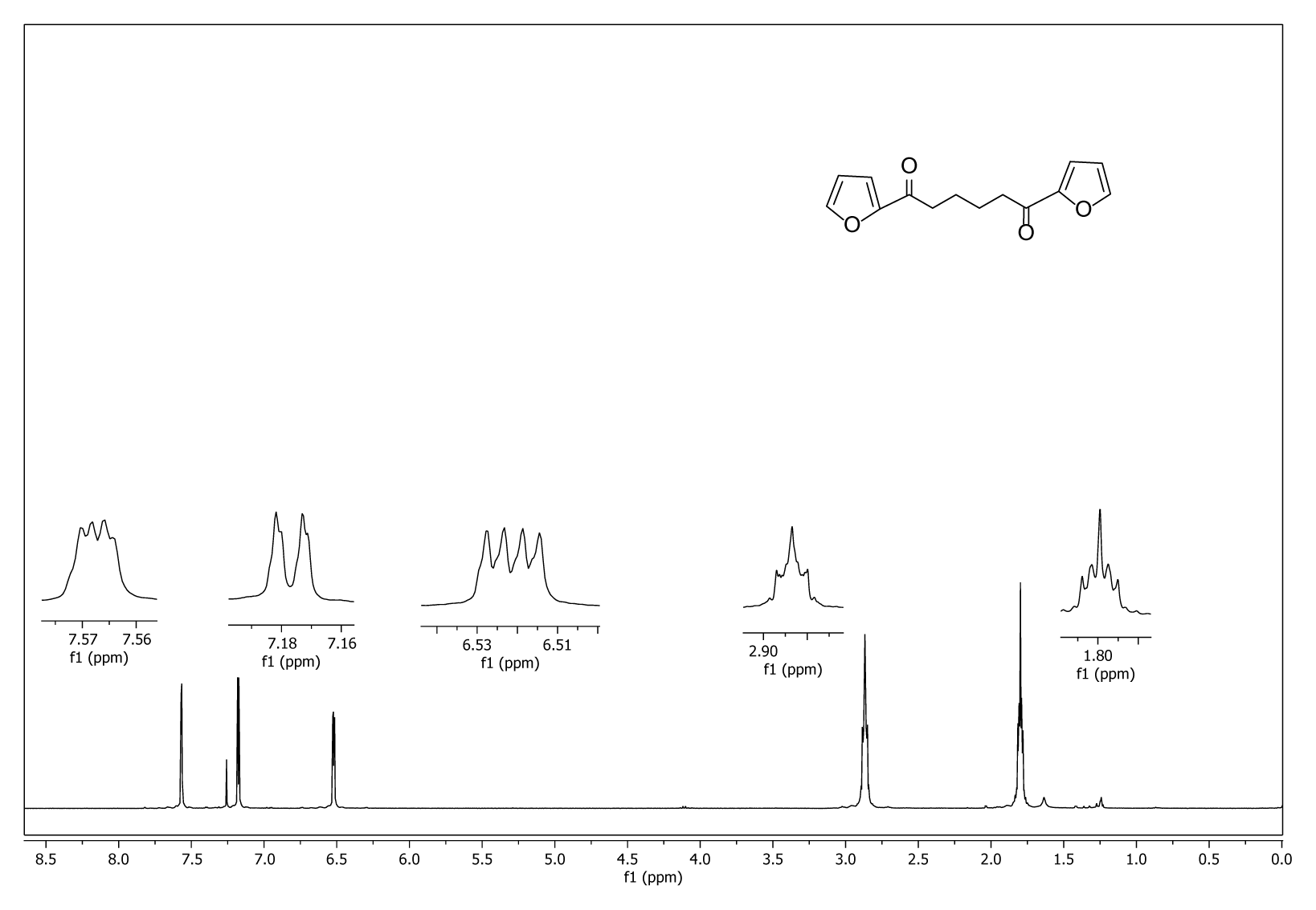

Supplement: Supplementary file 7 — 1H-NMR spectrum of the compound 11 (400 MHz, CDCl3). [file turkjchem-46-5-1397s7.tif]

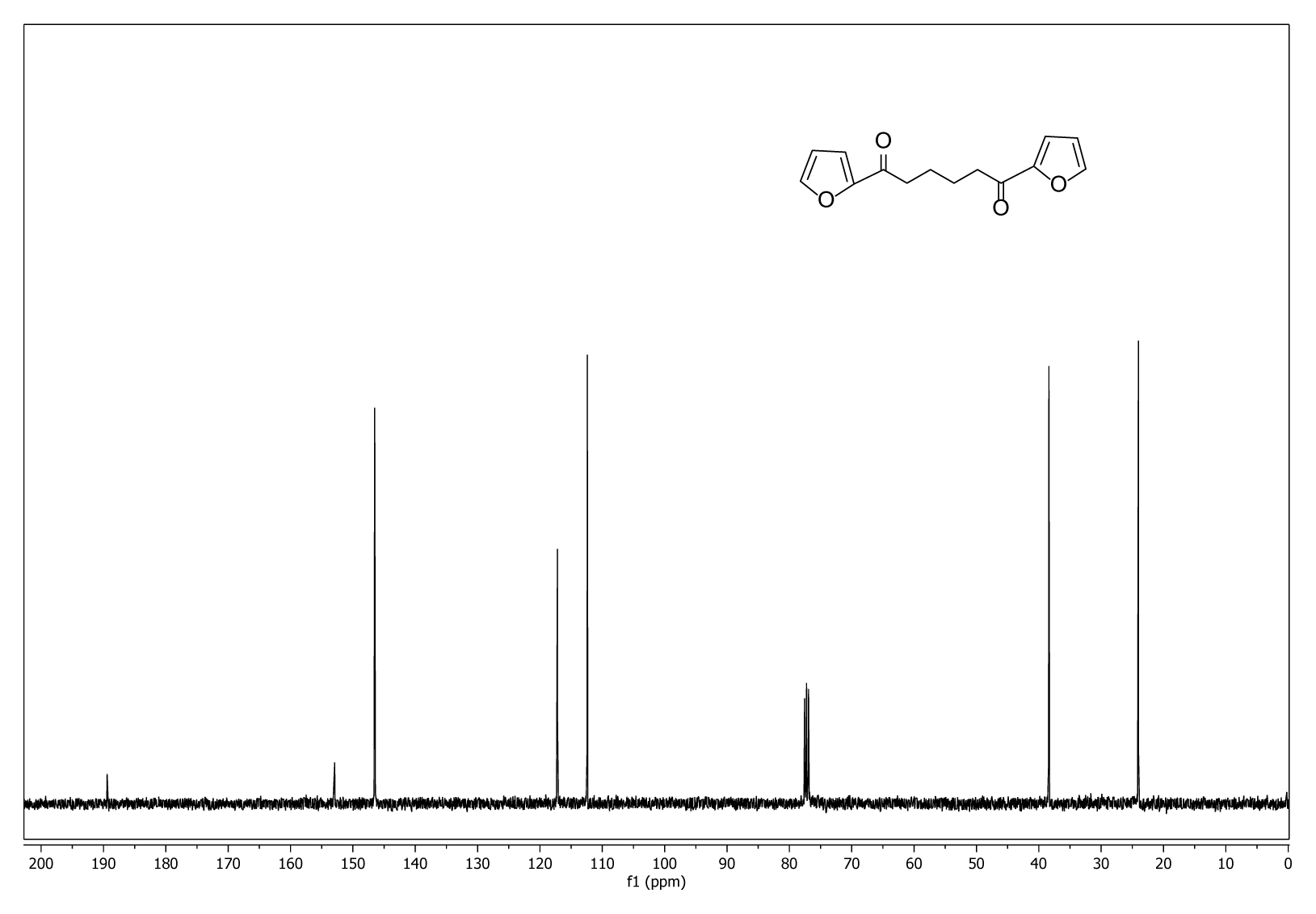

Supplement: Supplementary file 8 — 13C-NMR spectrum of the compound 11 (CDCl3, 100 MHz). [file turkjchem-46-5-1397s8.tif]

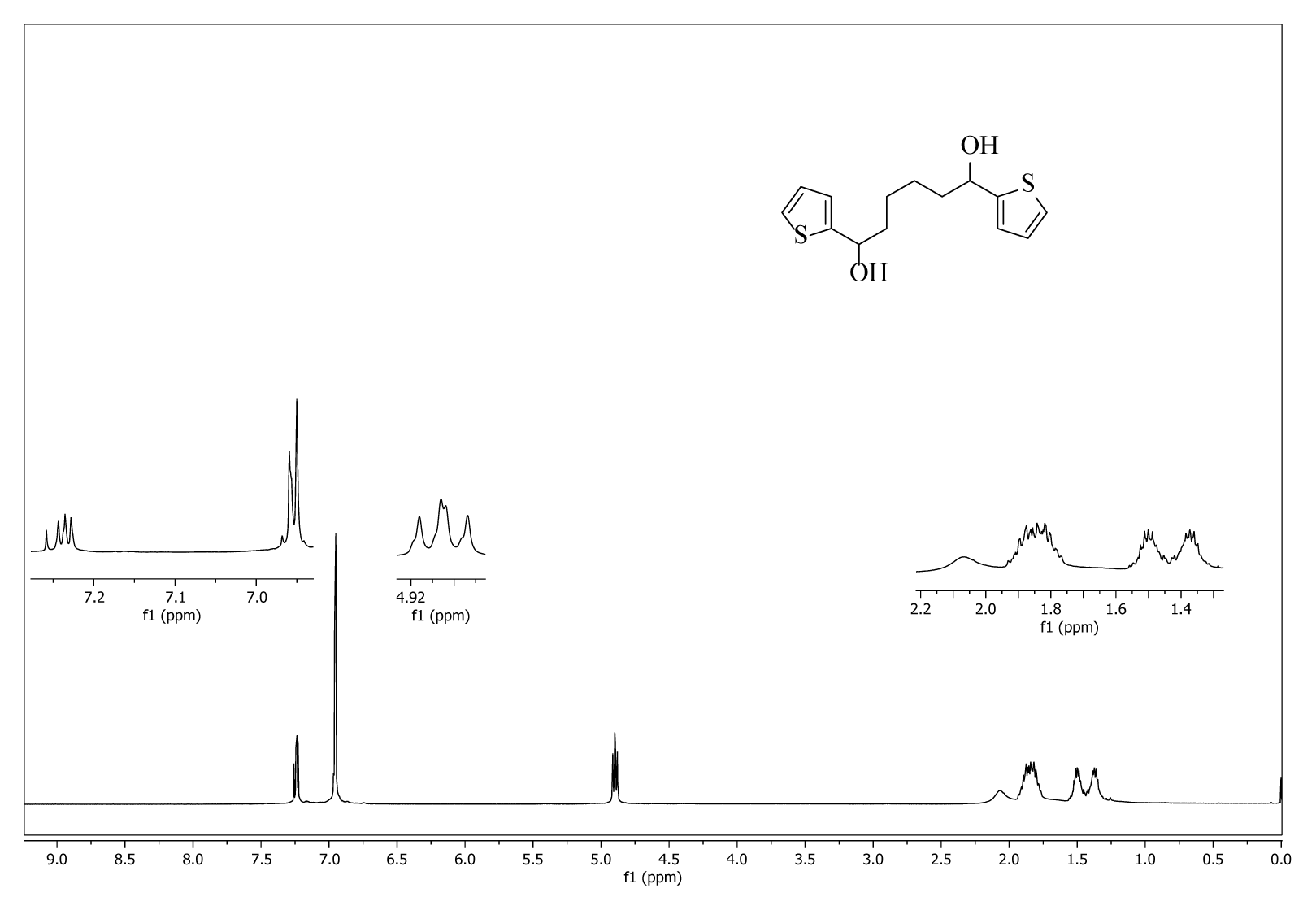

Supplement: Supplementary file 9 — 1H-NMR spectrum of diol 12 (400 MHz, CDCl3). [file turkjchem-46-5-1397s9.tif]

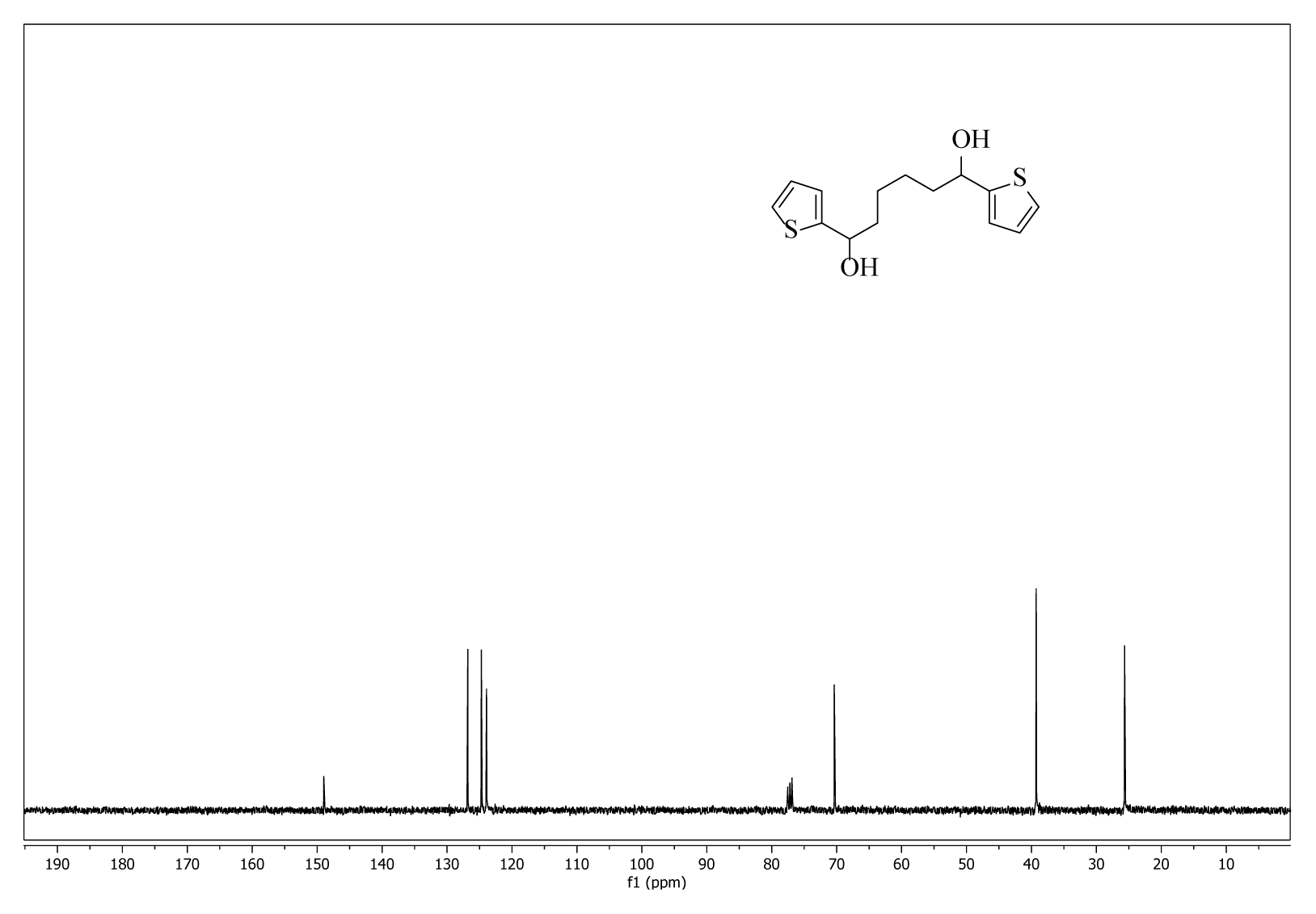

Supplement: Supplementary file 10 — 13C-NMR spectrum of diol 12 (CDCl3, 100 MHz). [file turkjchem-46-5-1397s10.tif]

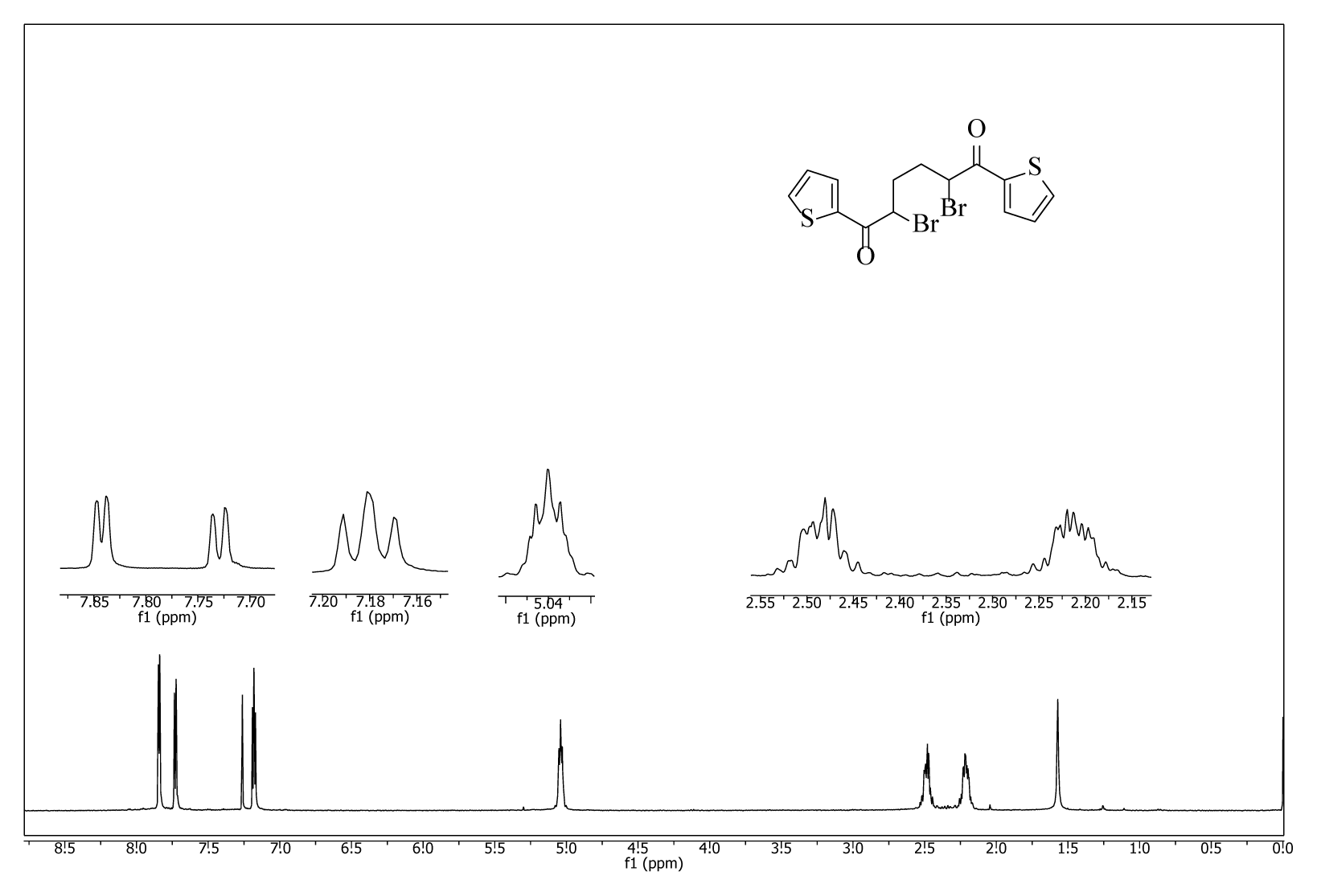

Supplement: Supplementary file 11 — 13C-NMR spectrum of dibromide 13 (CDCl3, 100 MHz). [file turkjchem-46-5-1397s11.tif]

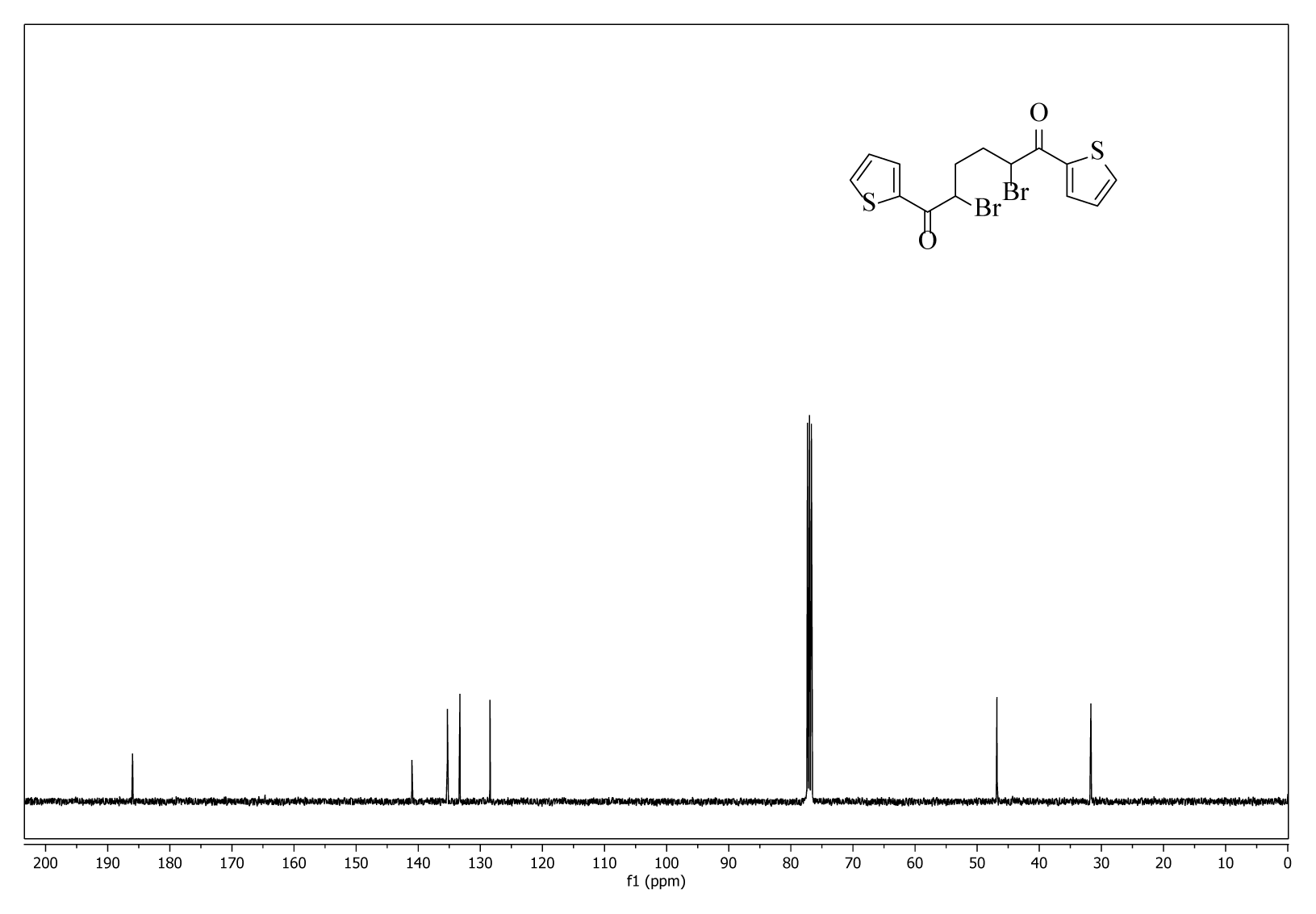

Supplement: Supplementary file 12 — 1H-NMR spectrum of dibromide 13 (400 MHz, CDCl3). [file turkjchem-46-5-1397s12.tif]

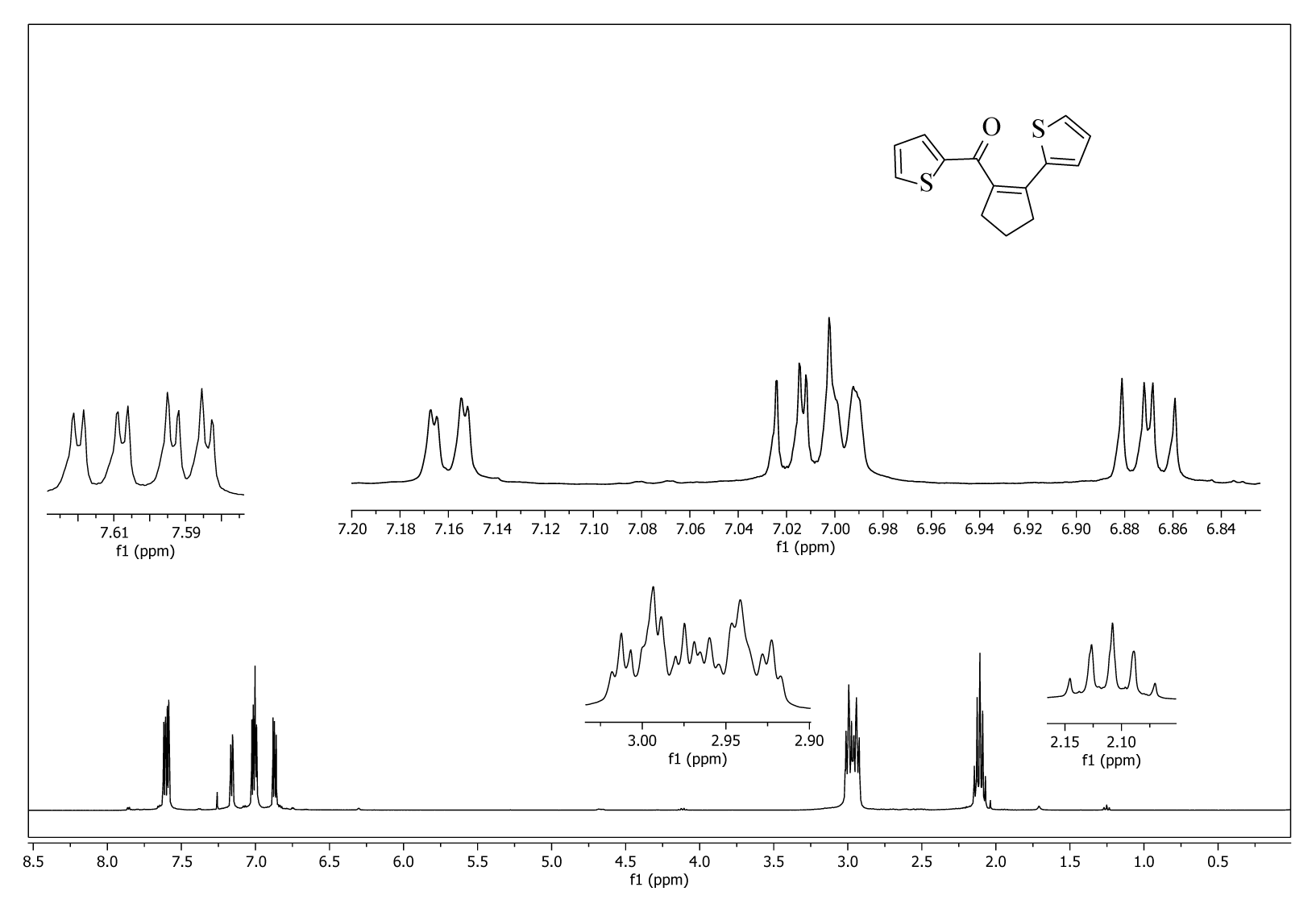

Supplement: Supplementary file 13 — 1H-NMR spectrum of the compound 14 (400 MHz, CDCl3). [file turkjchem-46-5-1397s13.tif]

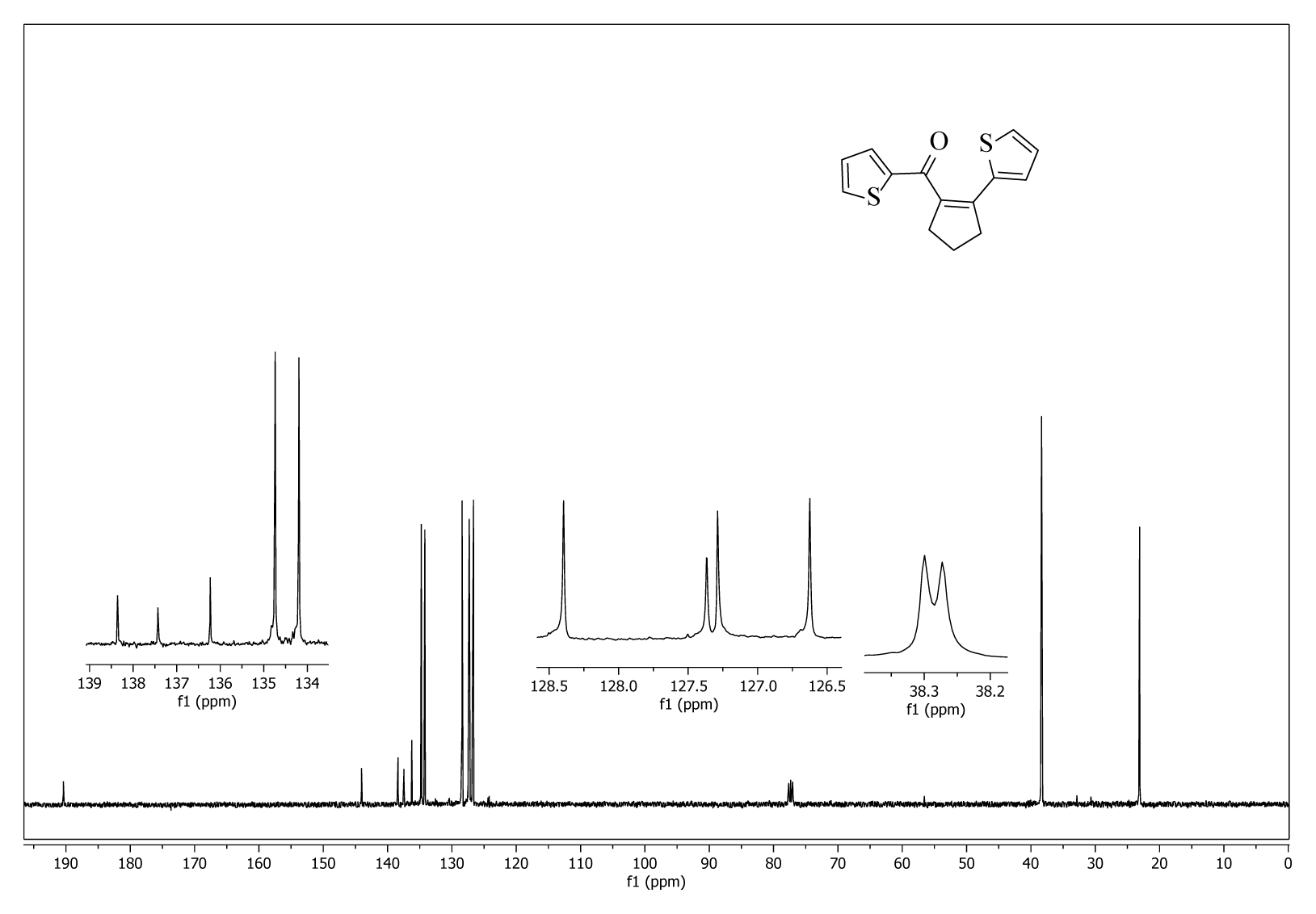

Supplement: Supplementary file 14 — 13C-NMR spectrum of the compound 14 (CDCl3, 100 MHz). [file turkjchem-46-5-1397s14.tif]

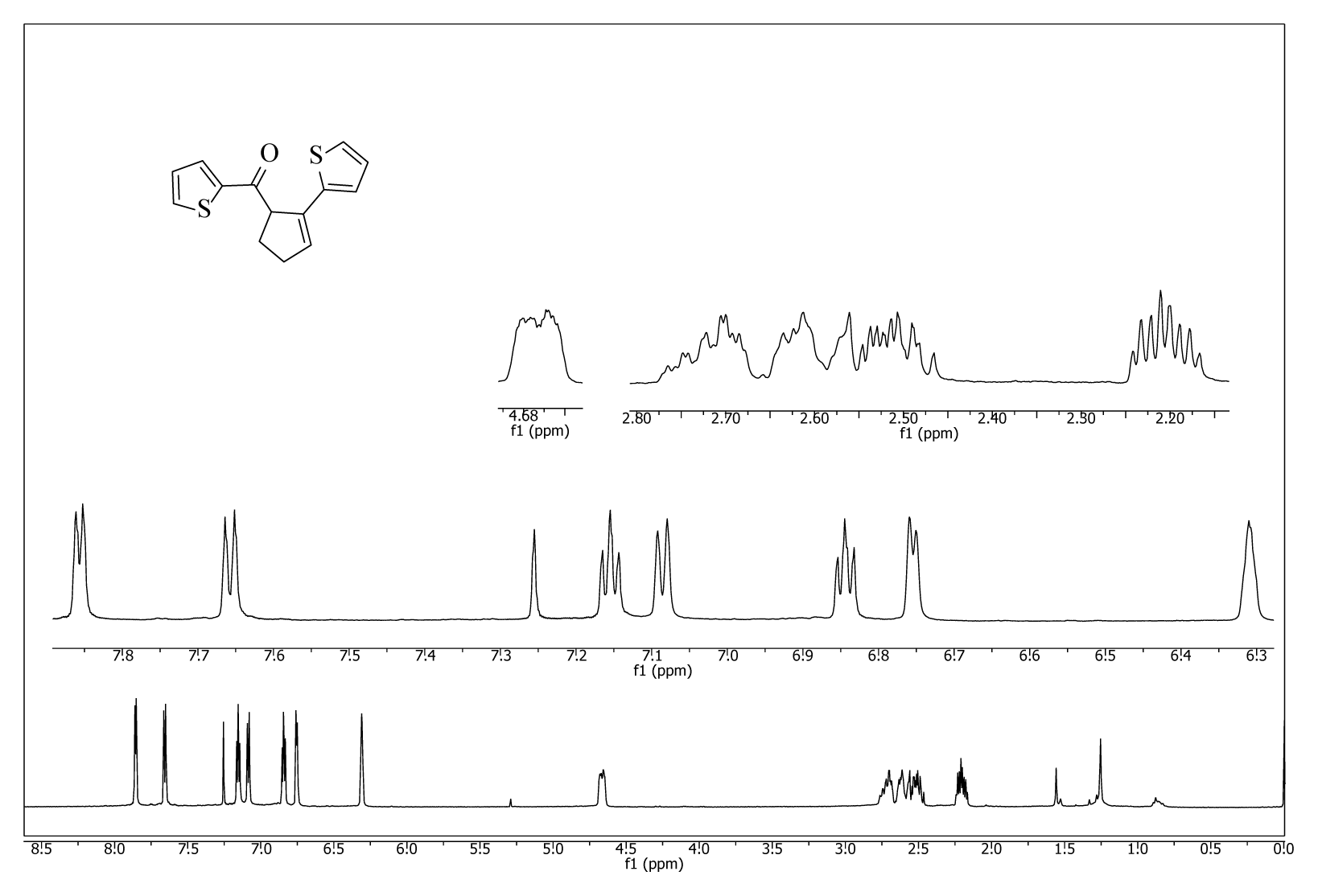

Supplement: Supplementary file 15 — 1H-NMR spectrum of the compound 15 (400 MHz, CDCl3). [file turkjchem-46-5-1397s15.tif]

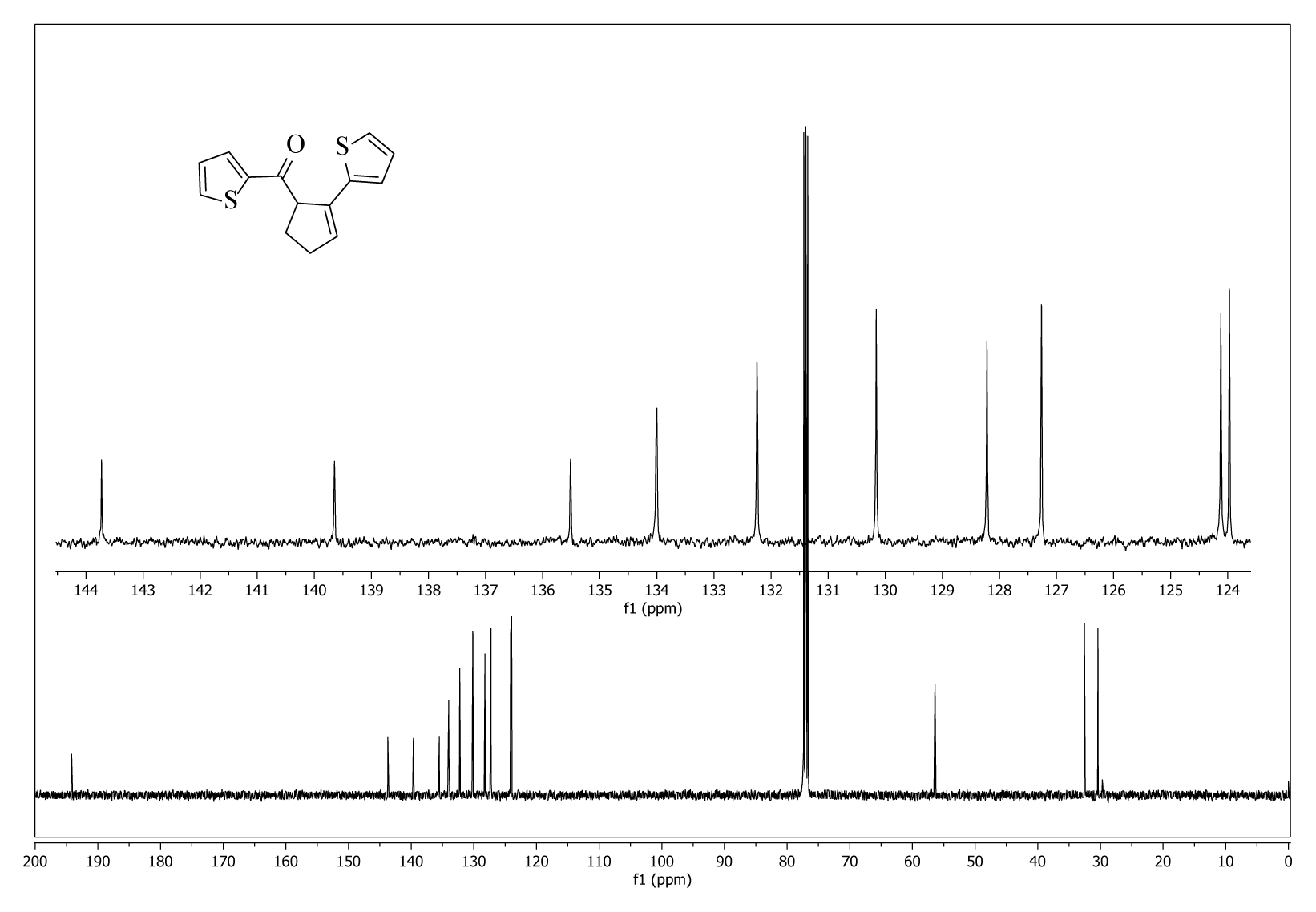

Supplement: Supplementary file 16 — 13C-NMR spectrum of the compound 15 (CDCl3, 100 MHz). [file turkjchem-46-5-1397s16.tif]

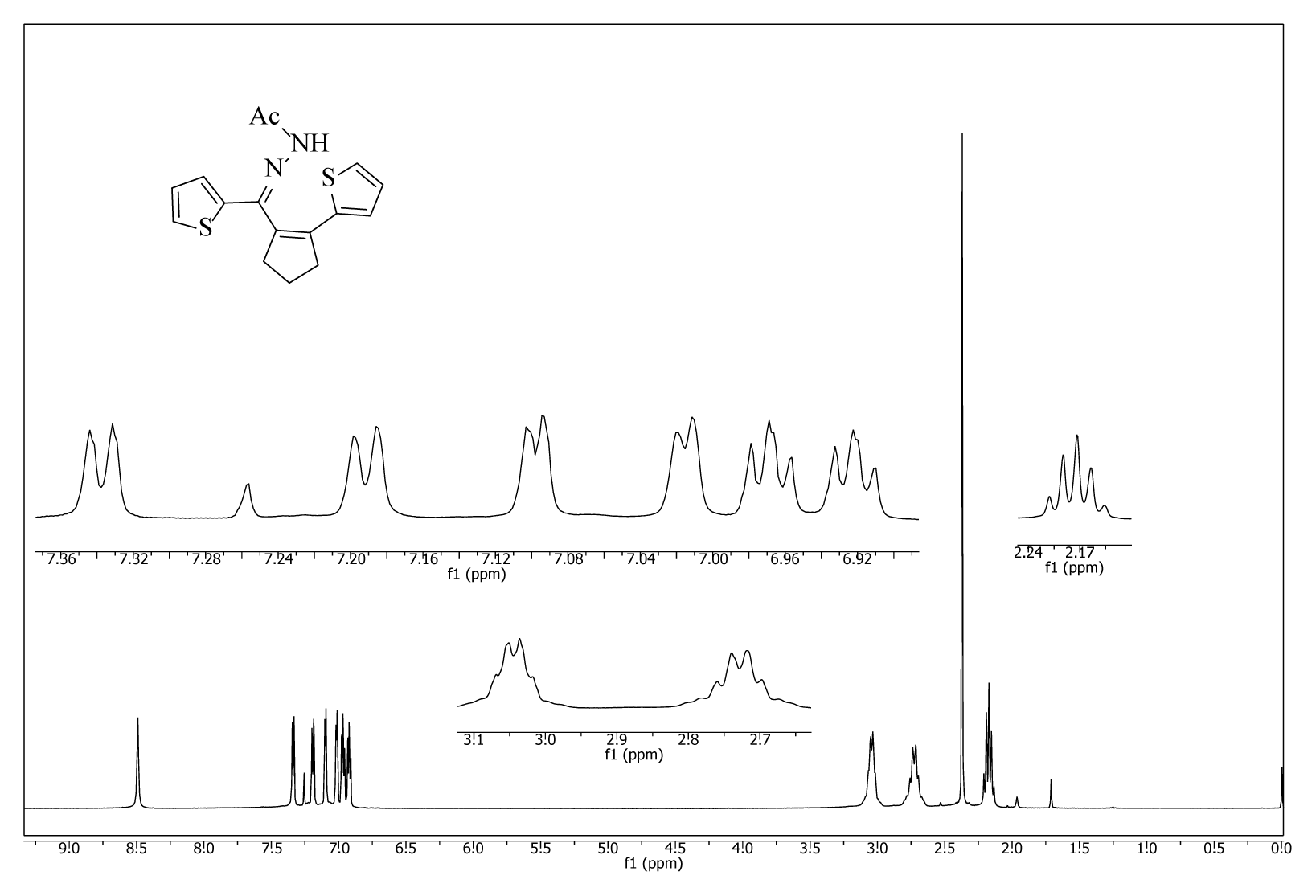

Supplement: Supplementary file 17 — 1H-NMR spectrum of the compound 16 (400 MHz, CDCl3). [file turkjchem-46-5-1397s17.tif]

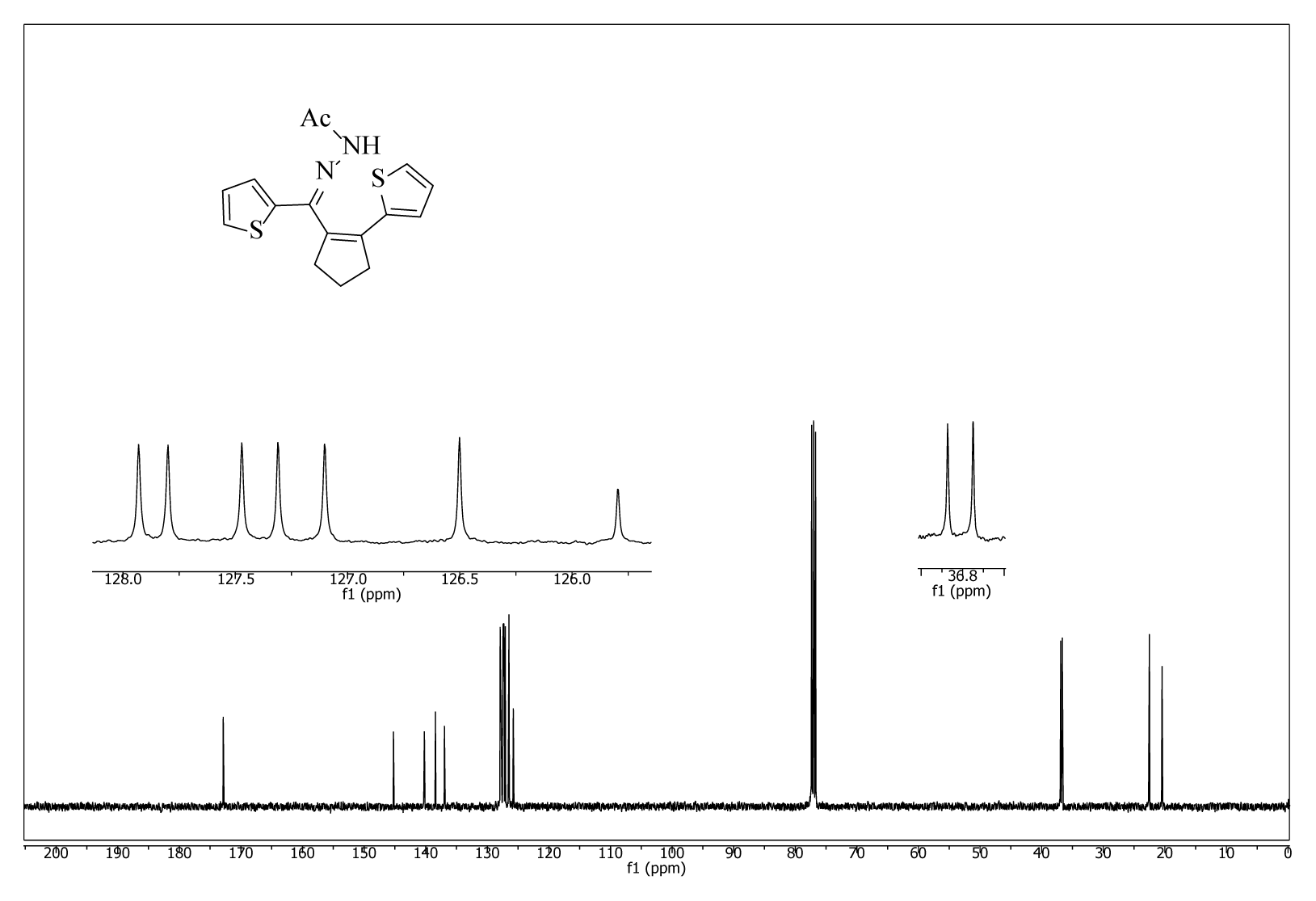

Supplement: Supplementary file 18 — 13C-NMR spectrum of the compound 16 (100 MHz, CDCl3). [file turkjchem-46-5-1397s18.tif]

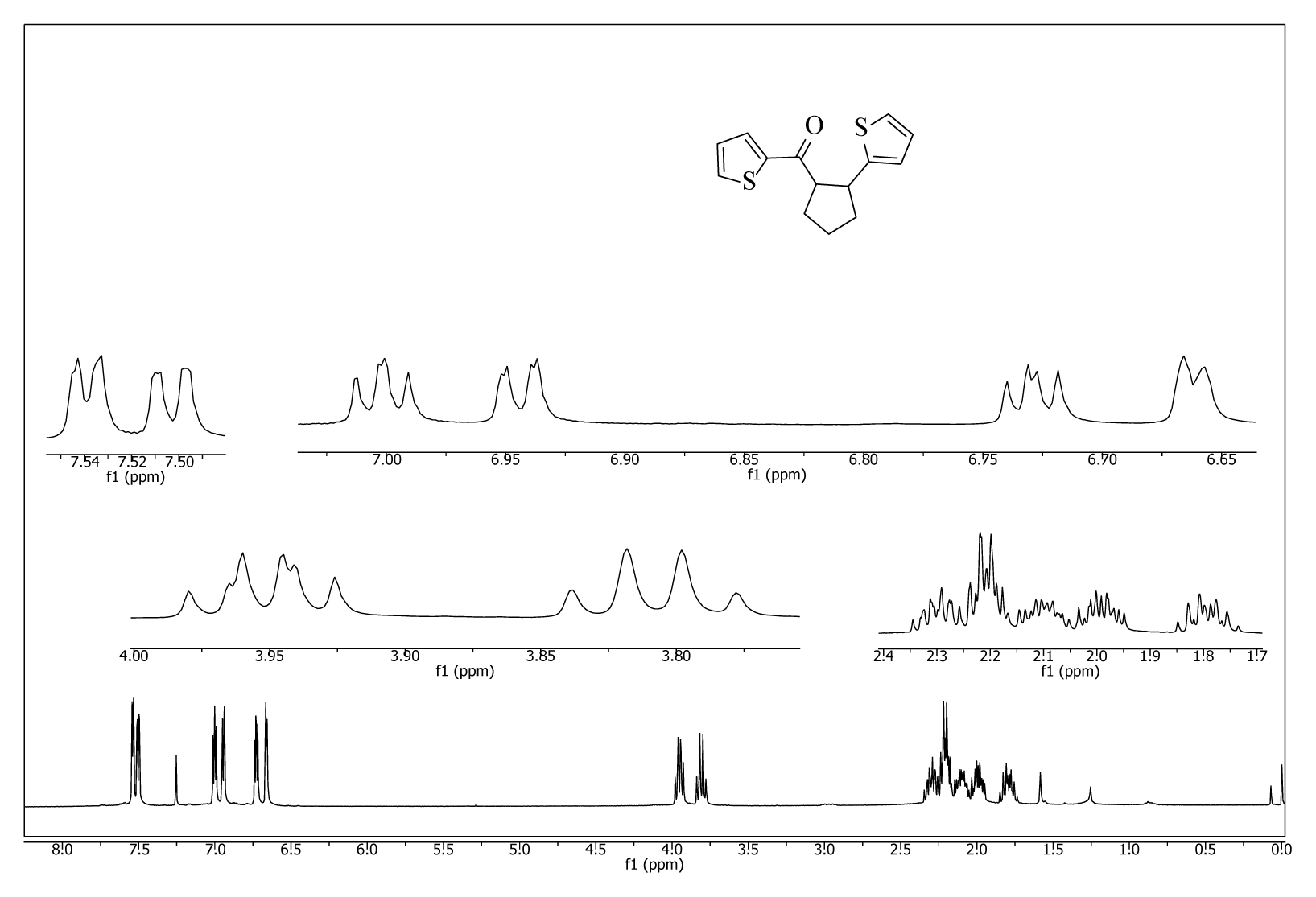

Supplement: Supplementary file 19 — 1H-NMR spectrum of the compound 17 (400 MHz, CDCl3). [file turkjchem-46-5-1397s19.tif]

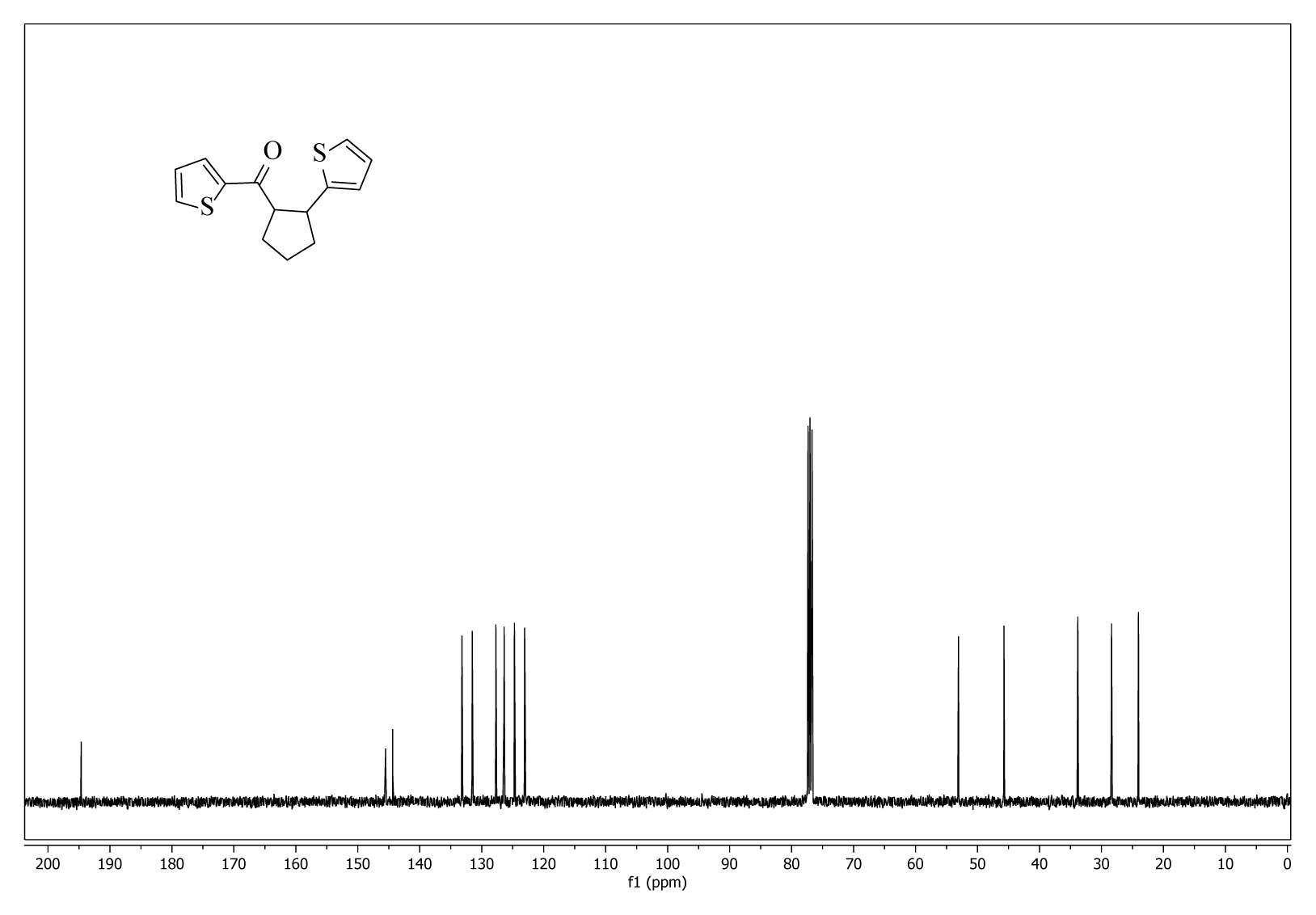

Supplement: Supplementary file 20 — 13C-NMR spectrum of the compound 17 (100 MHz, CDCl3). [file turkjchem-46-5-1397s20.tif]

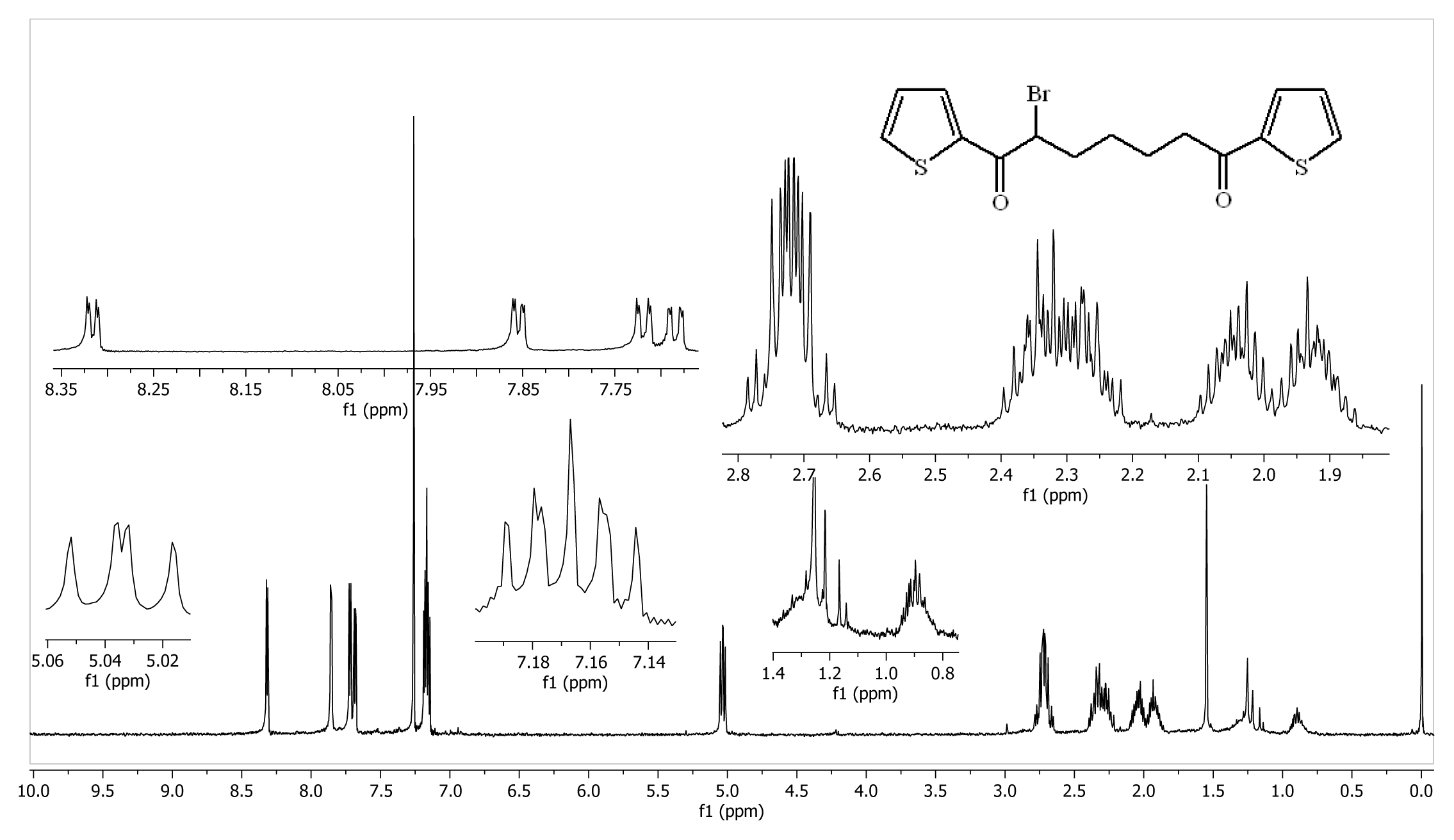

Supplement: Supplementary file 21 — 1H-NMR spectrum of the compound 18 (400 MHz, CDCl3). [file turkjchem-46-5-1397s21.tif]

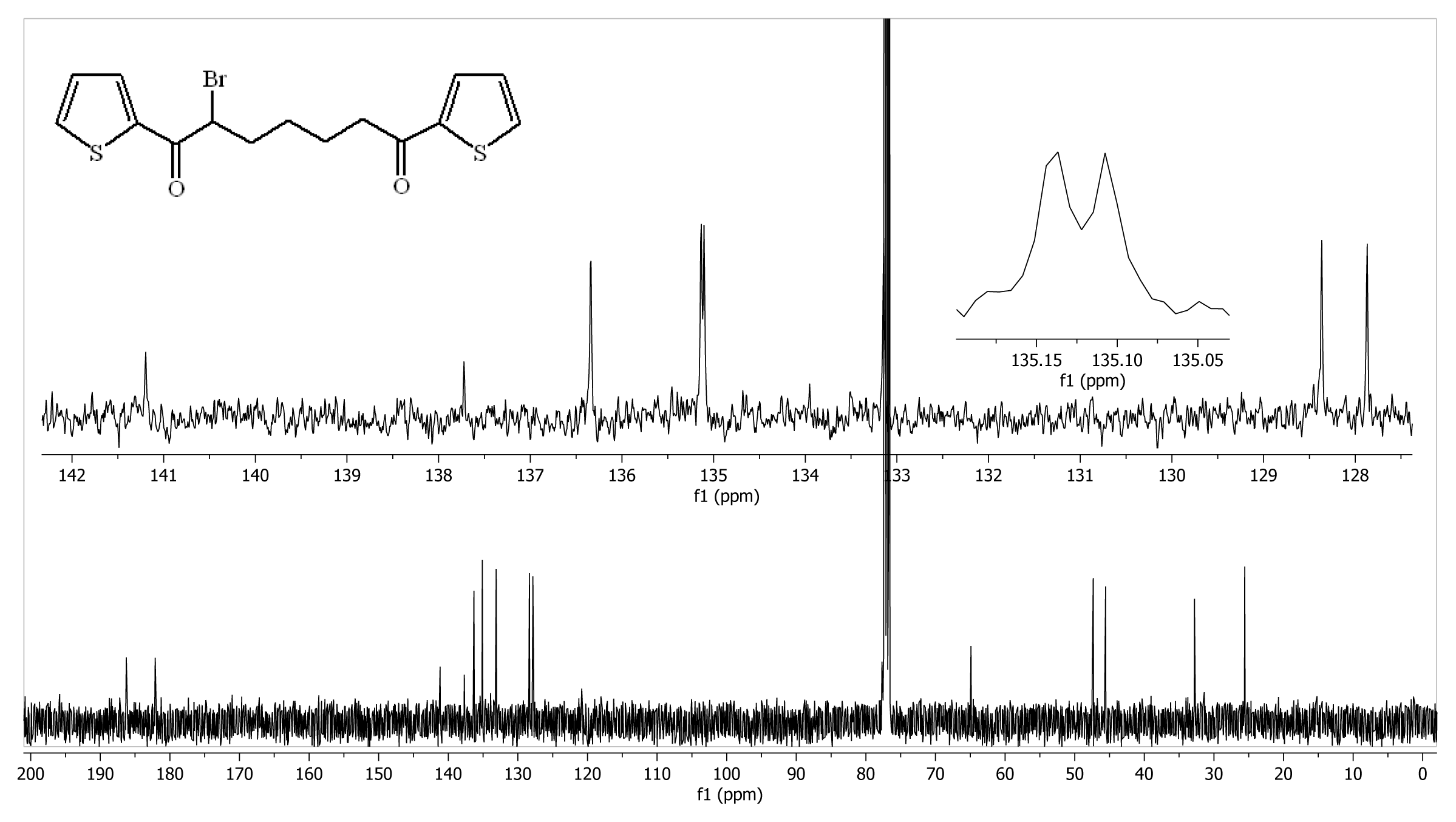

Supplement: Supplementary file 22 — 13C-NMR spectrum of the compound 18 (100 MHz, CDCl3). [file turkjchem-46-5-1397s22.tif]

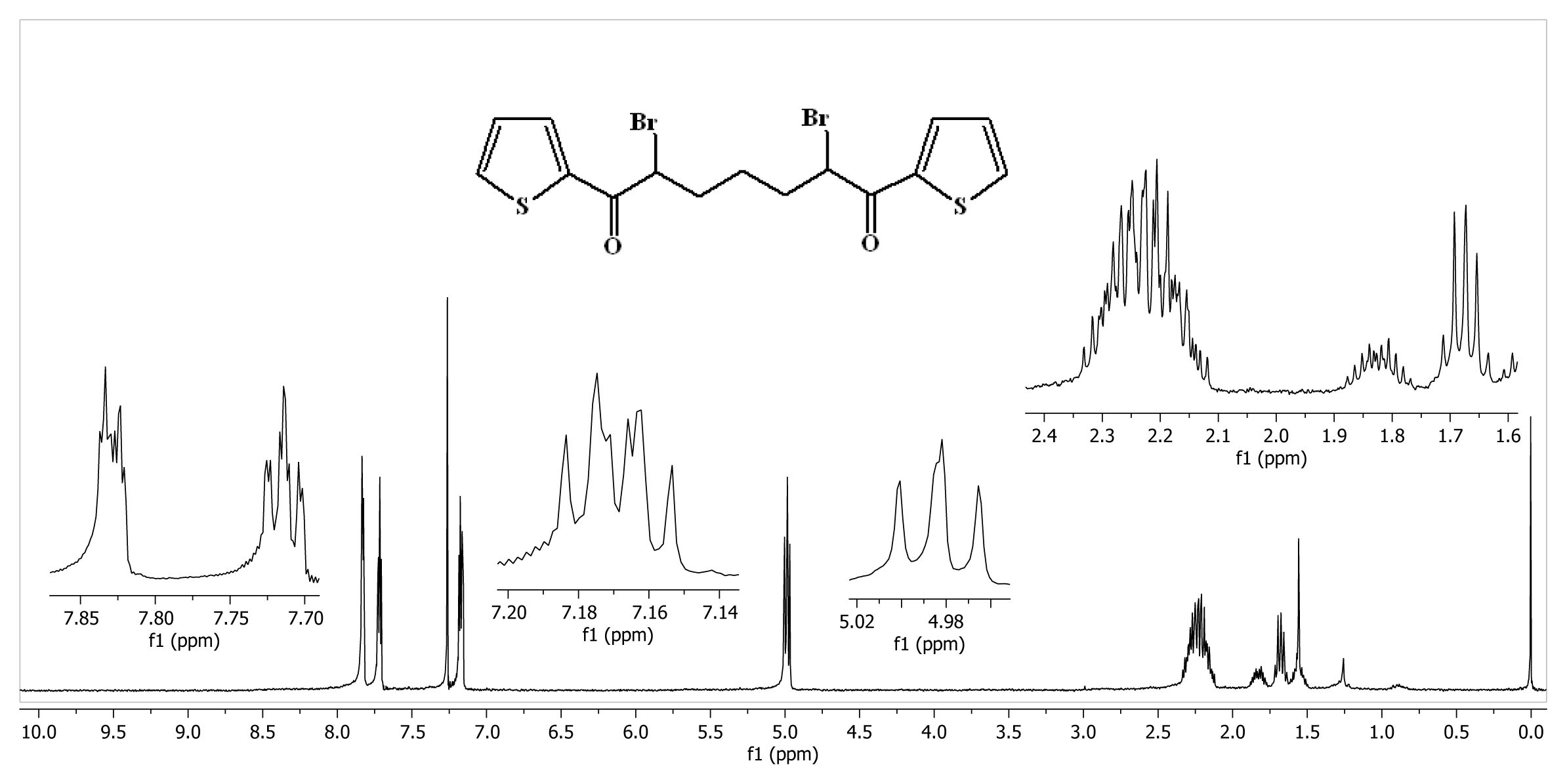

Supplement: Supplementary file 23 — 1H-NMR spectrum of diromide 19 (400 MHz, CDCl3). [file turkjchem-46-5-1397s23.tif]

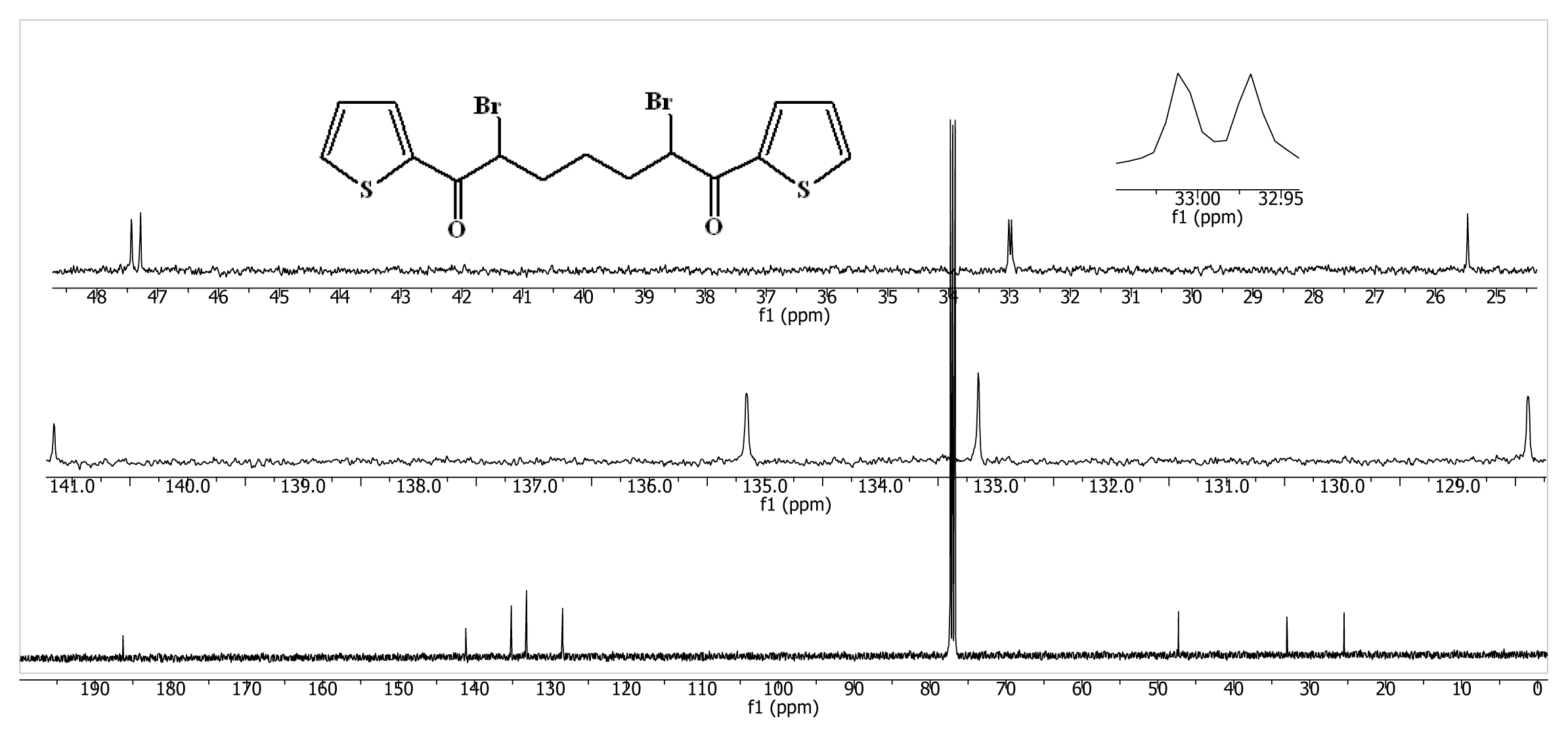

Supplement: Supplementary file 24 — 13C-NMR spectrum of diromide 19 (100 MHz, CDCl3). [file turkjchem-46-5-1397s24.tif]

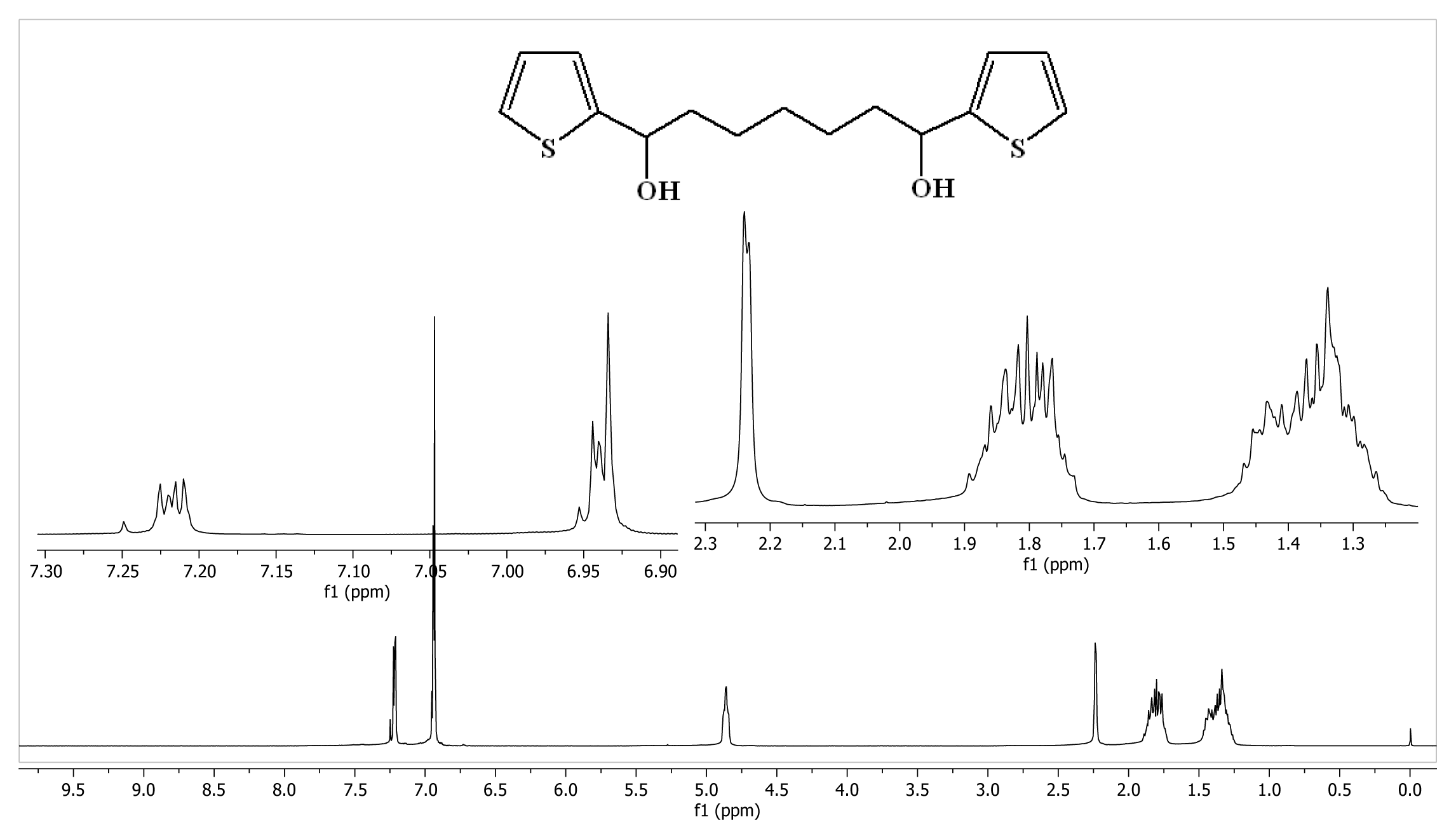

Supplement: Supplementary file 25 — 1H-NMR spectrum of diol 20 (400 MHz, CDCl3). [file turkjchem-46-5-1397s25.tif]

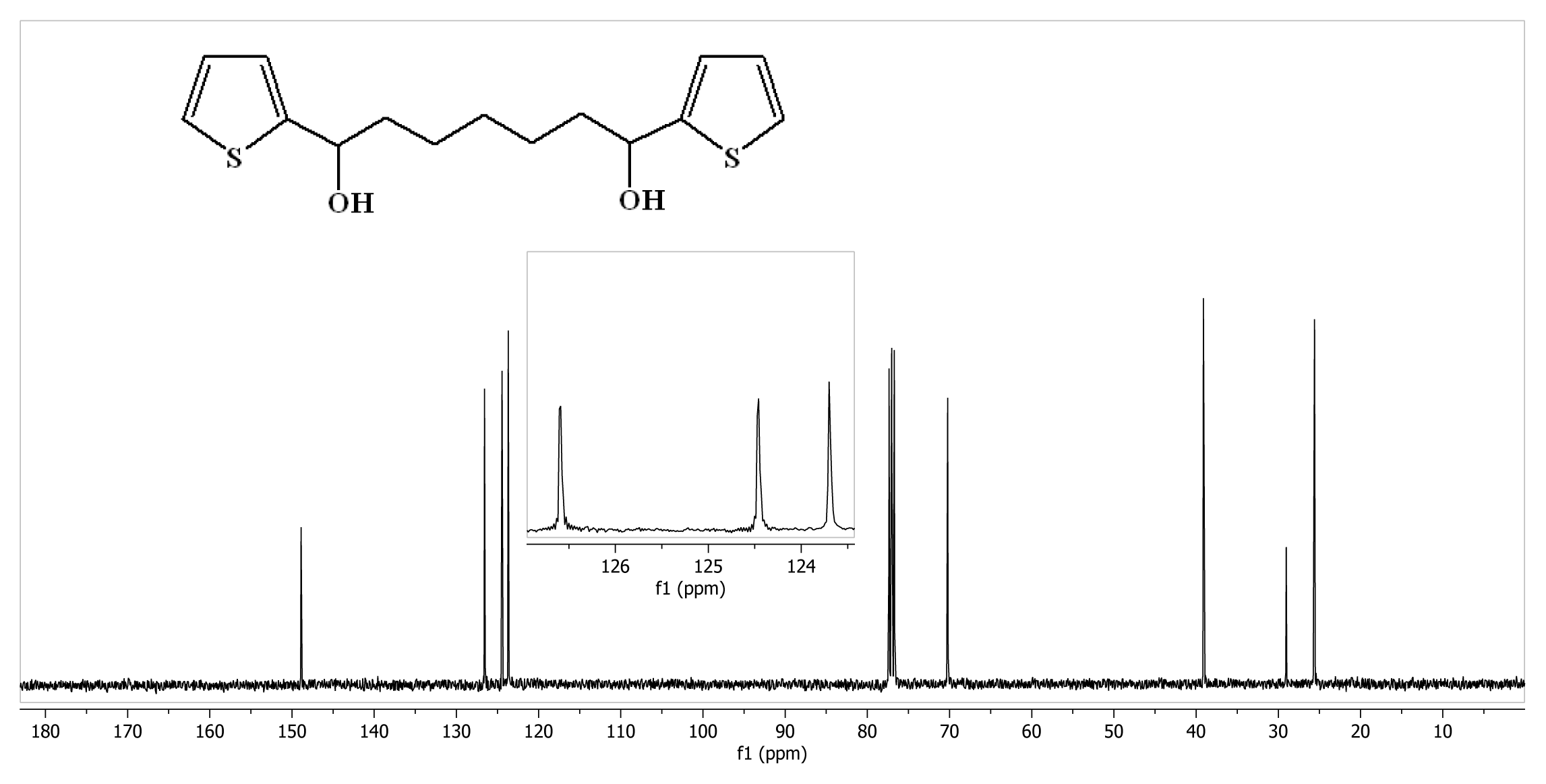

Supplement: Supplementary file 26 — 13C-NMR spectrum of diol 20 (100 MHz, CDCl3). [file turkjchem-46-5-1397s26.tif]

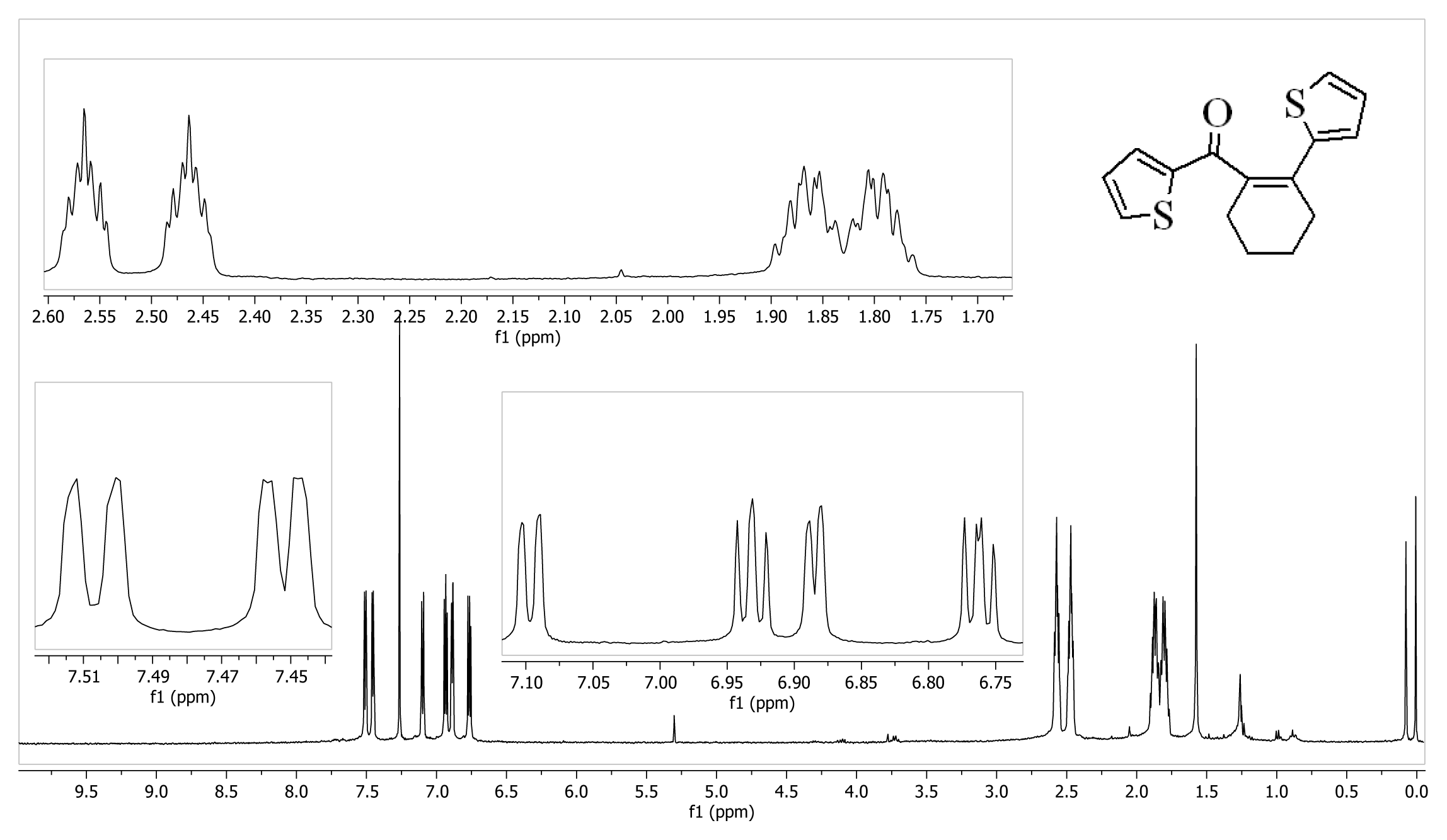

Supplement: Supplementary file 27 — 1H-NMR spectrum of the compound 21 (400 MHz, CDCl3). [file turkjchem-46-5-1397s27.tif]

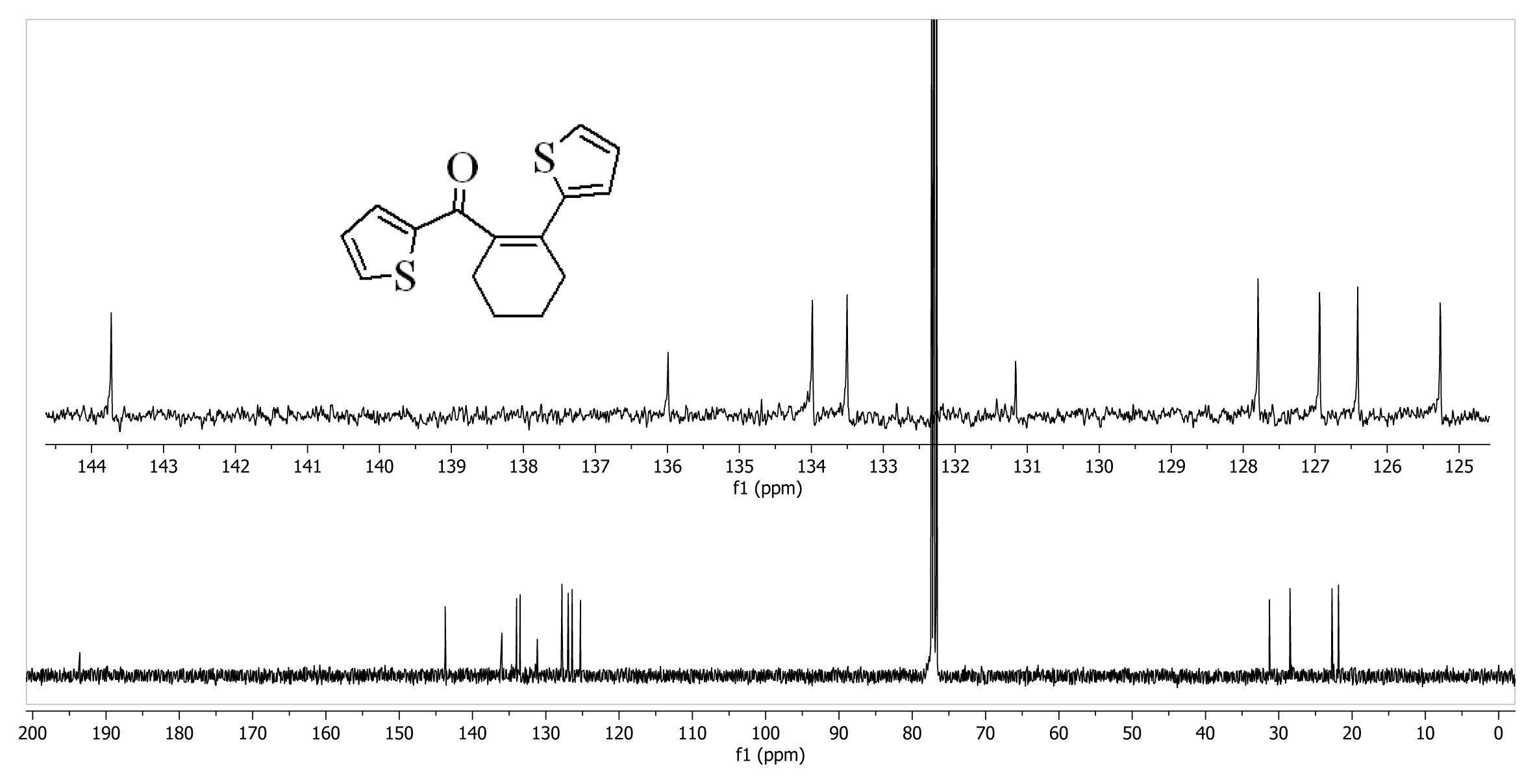

Supplement: Supplementary file 28 — 13C-NMR spectrum of the compound 21 (100 MHz, CDCl3). [file turkjchem-46-5-1397s28.tif]

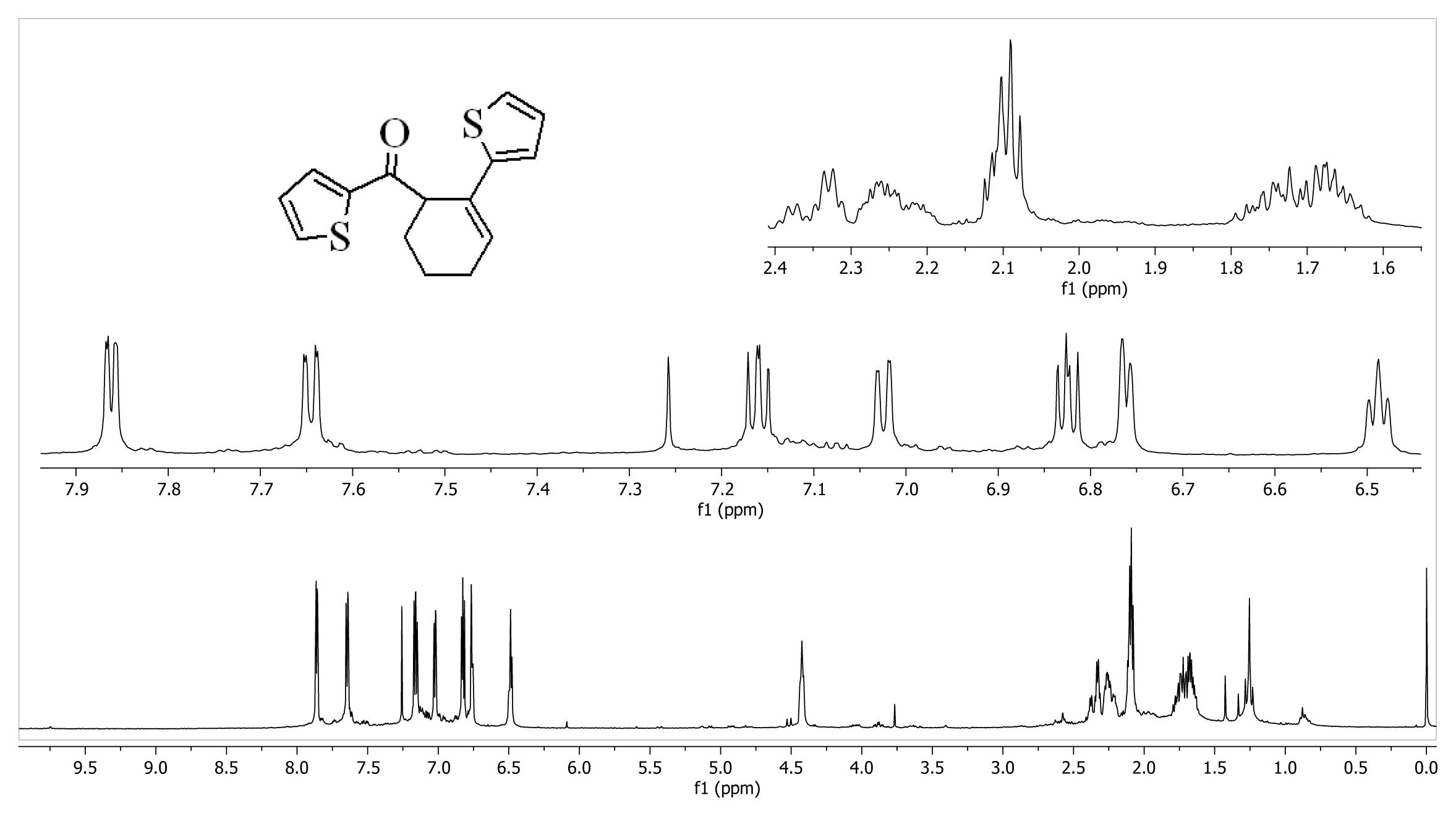

Supplement: Supplementary file 29 — 1H-NMR spectrum of the compound 22 (400 MHz, CDCl3). [file turkjchem-46-5-1397s29.tif]

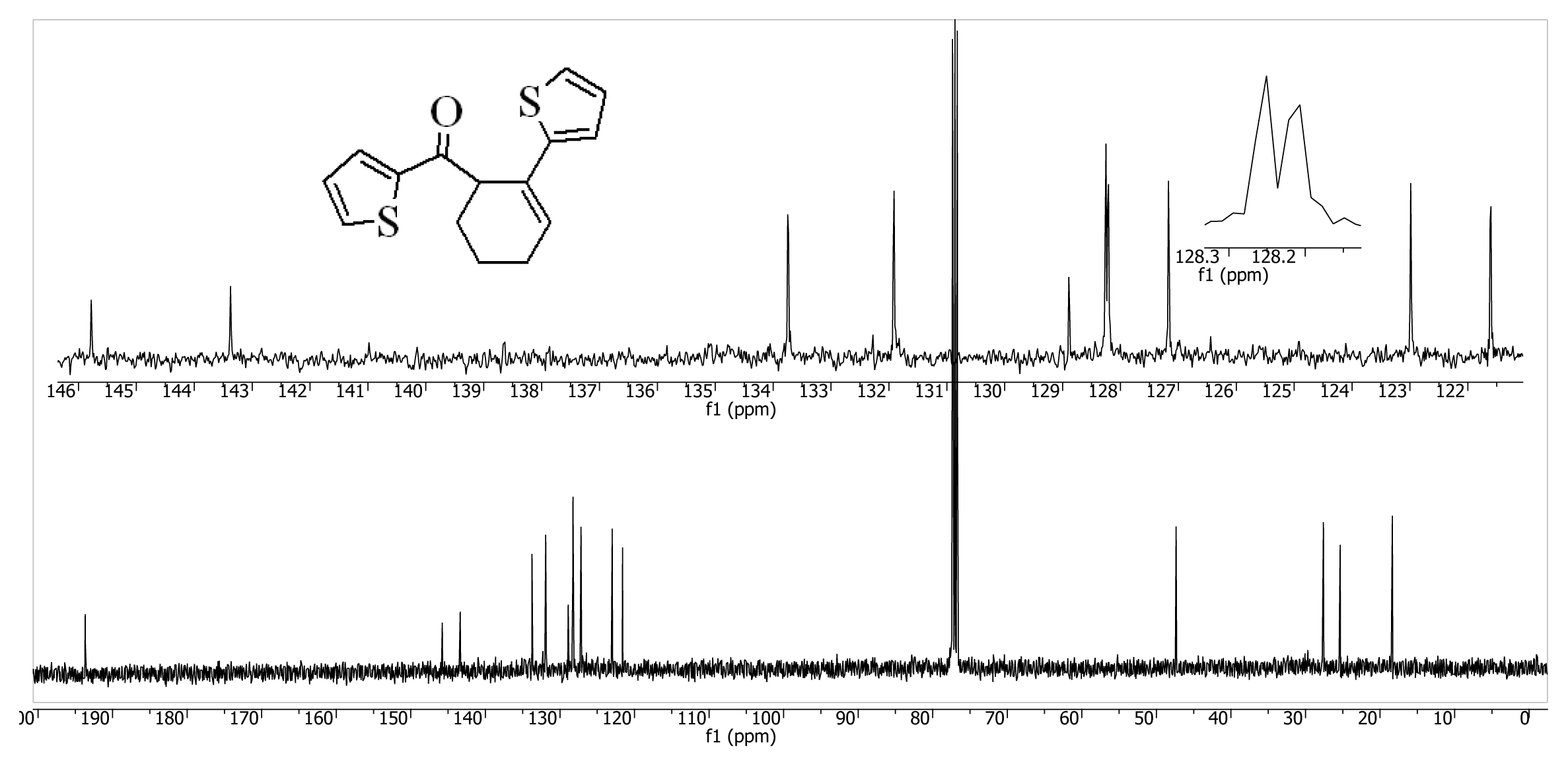

Supplement: Supplementary file 30 — 13C-NMR spectrum of the compound 22 (100 MHz, CDCl3). [file turkjchem-46-5-1397s30.tif]
